# Survey about Barriers and Solutions for Enhancing
 Computational Reproducibility in Scientific Research

**DOI:** 10.12688/f1000research.172013.1

**Published:** 2025-11-18

**Authors:** Yuri Andrei Gelsleichter, Rita Banzi, Florian Naudet, Constant Vinatier, István Kertész, Monika Varga

**Affiliations:** 1 Department of Soil Science, Institute of Environmental Sciences, Hungarian University of Agriculture and Life Sciences, Gödöllő, 2100, Hungary; 2 Laboratory for Health Regulatory Policies, Mario Negri Institute for Pharmacological Research IRCCS, Milan, Italy; 3 CHU Rennes, Inserm, Irset (Institut de recherche en santé, environnement et travail)-UMR_S 1085, CIC 1414 (Centre of Clinical Investigation of Rennes), University of Rennes, Rennes, France; 4 Institut Universitaire de France, Paris, France; 5 Institute of Food Science and Technology, Hungarian University of Agriculture and Life Sciences, Budapest, 1118, Hungary; 6 Institute of Animal Sciences, Hungarian University of Agriculture and Life Sciences, Kaposvar, 7400, Hungary

**Keywords:** Open science; Open Data; Open Code; Research transparency; Science integrity; Open Survey; Reproducibility

## Abstract

**Background:**

Rapid development and adoption of digital technologies across all research disciplines underlines the need for accessible and reusable computational data and code.

**Methods:**

An anonymous, multidisciplinary survey covering open science, data publishing and reuse, as well as code publishing and reuse was conducted to gather insights into researchers’ practices, needs, and barriers.

**Results:**

A total of 254 people initiated the survey, with 133 complete responses (mostly from Europe, equally distributed among scientific fields). Survey revealed that registered reports, replication studies and pre-registration are the least applied practices (52%, 38% and 42%), while open software and OA publishing demonstrated widespread adoption (83% and 69%) of the respondents, respectively. Data sharing is hindered mostly by *lack of time* (60%) and *sufficient funding* (44%). Among the predefined obstacles of code sharing, again, the *lack of time to build proper documentation* (65%), *pressure to publish* (51%), and the *insufficient funding* (42%) are the most mentioned reasons. On the other hand, most stimulating factors are the requirement of journals to share data/codes (score: 482), followed by incentives and rewards by institutions (score: 439). The survey showed that 28% of researchers never tried to reproduce a study, and when replication was attempted, researchers often found that open data (70%), open code (71%), and metadata (86%) were missing or incomplete. The analysis of open-ended responses highlighted the need for training, career-stage guidelines, and basic programming skills for researchers.

**Conclusions:**

Although the likely low response rate may limit its representativeness, this study provides an up-to-date snapshot. A recurrent theme throughout the responses is the need for structural incentives and institutional support. Researchers claim that making work reproducible requires time, resources, and expertise; however, these efforts are rarely rewarded in conventional academic evaluation systems, highlighting the need for a systemic cultural shift.

## Introduction

Reproducibility is crucial to scientific integrity and credibility, as it helps to verify how research data are generated and identify data manipulation or methodological flaws ( National Academies of Sciences, Engineering, and Medicine, 2019). Utilizers of scientific results (e.g., broader scientific communities, policymakers, clinicians, and the wider public) rely on scientific findings. If the results are not reproducible, it undermines trust and might lead to poor decisions in several areas, such as health, economics, and environmental regulation. Several studies have focused on unsuccessful replication attempts ( Begley & Ellis, 2012; Ioannidis, 2005; Munafò et al., 2017; Open Science Collaboration, 2015; The Brazilian Reproducibility Initiative et al., 2025) that increasingly brought the crisis narrative to the fore. Reproducibility is an important precondition and cornerstone of research quality ( National Academies of Sciences, Engineering, and Medicine, 2019), and has been widely discussed across various disciplines in the past decade, including psychology, medicine, economics, and biology ( Begley & Ellis, 2012; Ioannidis, 2005; Open Science Collaboration, 2015).

Currently, computational reproducibility has become critically important, as much of today’s research work relies heavily on digitalized data and computational tools throughout the entire research life cycle from research design, through computer-aided data analysis, to automated reporting tools. Computational reproducibility can be defined as “the ability to recreate results using the original data and code (or at least a detailed description of the analyses)” ( Crüwell et al., 2023), and, computational scientist as “an academic whose research has both code and data components” ( Stodden, 2010). This concept holds that “when you use the same data as in the published article, you can reproduce the same results” ( Lakens, 2022), “the data and the computer code used to analyze the data be made available to others” ( Peng, 2011), thereby enabling the evaluation and reuse of research outputs, data, and code by other researchers. Research code or research software, defined as “Software that is used to generate, process or analyse results that you intend to appear in a publication” by ( Hettrick et al., 2014), is crucial for the reproducibility of research. Commonly refers to case-specifically developed computer code, from a few lines to a professional package, used in the process of scientific or academic research to analyze data, simulate models, or process information. Therefore, the sharing and evaluation of research codes are of key importance.

In recent years, a few survey-based studies have been conducted on reproducibility ( Baker, 2016), data management and sharing ( Tenopir et al., 2011; Van Den Eynden et al., 2016), computational reproducibility ( AlNoamany & Borghi, 2018; Stodden, 2010), as well as on discipline-related specificities, for example, computational reproducibility in computational biology ( Barone et al., 2017) and geosciences ( Reinecke et al., 2022). In terms of code and data sharing, the most comprehensive survey on computational reproducibility was prepared five years ago ( AlNoamany & Borghi, 2018).

In current scientific research, repleting with new digital tools and technologies in all disciplines, there is an emerging need for data and code reuse. Therefore, this study aimed to obtain an up-to-date overview that captures the current landscape of researchers’ needs, barriers, and practices across various disciplines, with the goal of improving scientific reproducibility.

Accordingly, a survey-based study was conducted to address the following key research questions:

1. What are the main perceptions about practices that support computational reproducibility?
2. How are code and data shared during the publication?
3. What obstacles impede computational reproducibility in the practices of researchers?
4. Which methods and tools are frequently employed to support computational reproducibility?
5. How often do researchers attempt to replicate the studies of their peers, and how do they succeed?
6. How do the responses to the above questions vary based on career stage, academic discipline, geographical location, and related factors?

## Material and methods

The present study followed the Checklist for Reporting Results of Internet E-Surveys (CHERRIES) guidelines ( Eysenbach, 2004) for reporting results of internet e-surveys and reflects on all of its necessary elements in the current Method section. The study protocol was registered on the Open Science Framework (OSF) prior to data collection ( Gelsleichter et al., 2024). The original survey material, anonymized survey responses, and descriptive statistical analysis results are publicly available in ( Gelsleichter et al., 2025).

### Survey design

To provide meaningful insights into Open Science (OS) practices and computational reproducibility, we considered an anonymous open survey to be the most suitable approach. This enables us to explore general awareness and individual attitudes, which are difficult to assess through direct observation or literature review. Survey questions were developed in several rounds, in consultation with members of our consortium OSIRIS (Open Science to Improve Reproducibility in Science, funded by the European Union under the grant agreement 101094725), resulting in six sections with 35 questions, along 12 pages: demographic questions (6 questions), open science practices, supporting computational reproducibility (8 questions), data publishing (6 questions), data reuse (1 question), tools and code publishing (12 questions), and code reuse (2 questions). The first technical section of the survey aimed to assess the awareness and extent of the use of practices supporting open science and computational reproducibility. A complete survey with the questions and study protocol is provided in the OSF ( Gelsleichter et al., 2024).

The survey was conducted online using the LimeSurvey, a free and open-source software ( LimeSurvey, 2025). Instead of sending the survey directly to each participant, it was shared on social media channels (further details are provided in the next section). This survey was designed with the possibility of breaking down or filtering answers according to screening questions (i.e., filled out by researchers, carrying out quantitative research). Since we did not have control over participants’ invitation, a concern was raised about the software’s technical level of participants; for example, if the survey becomes too complex at some point, they could answer improperly (just to move on) or drop the survey. To avoid this, skipping mechanisms were set in some questions, based on previous ones; for example, in the question of *Choose the characteristics that describe the kind of research data you generate;* if the participant responds that ***I do not produce data in my research**,* the system skipped ahead to the next section. This mechanism was implemented in ten questions, the following: 1.4, 2.7, 3.1, 3.2, 3.3, 4.1, 5.4, 5.5, 5.8, and 6.1. The respondents were able to edit their responses as backward navigation was enabled. Furthermore, because the survey was comprehensive and long, multiple fillings were not considered as an issue, so a checking mechanism was not implemented. To avoid personal data collection, IP checks and the use of cookies were not performed.

To give the participant the necessary background of certain (more complex/technical) items, aiming to avoid any skipping, most of the items carried explanatory ‘tooltip’ style messages along the survey, similar procedure as ( Reinecke et al., 2022). Before conducting the survey, rounds of internal pre-pilot testing were carried out to provide feedback about technical usability, clarity of questions, and time requirement to complete the survey, involving OSIRIS colleagues and PhD students. Feedback was taken into account, often suggesting editions, simplifications, and sometimes the inclusion of new questions. A similar approach was done by ( Stodden, 2010). The survey was conducted between 2024-02-23 and 2024-09-30.

### Survey population and recruitment

In line with the registered protocol, the survey targeted researchers regardless of their discipline, who collected quantitative (usually digital) data and analyzed and utilized them with computational methods and tools, to provide a general overview of attitudes, barriers, and practices in terms of computational reproducibility. In our interpretation, quantitative researchers, regardless of discipline, collect and use quantitative (usually digital) data and analyze and utilize them with computational methods and tools. Through this interpretation, various fields from the natural, applied, and social sciences are involved.

To reach out to a wide range of researchers in terms of geographic and scientific coverage, the survey link was shared via the official social media channels of OSIRIS (LinkedIn, X) and was re-shared by OSIRIS partners via flyers at scientific conferences ( iEMSs, 2024 and local events of Hungarian University of Agriculture and Life Sciences) and through blog posts on scientific community websites ( International Environmental Modelling and Software Society, 2024; Springer Nature Research Communities, 2024). A similar approach was used by ( AlNoamany & Borghi, 2018). The survey distribution relied on volunteer sampling within the research community, which eliminated the need to maintain a sensitive database of names and email addresses, which would be necessary for direct email-based recruitment.

### Ethics and consent

The survey-related work did not contain any research study on humans (individuals, samples or data). Since it is about research practices and workflows in context of computational reproducibility, and study was designed as an anonymous one, not involving human subjects in a sensitive or identifiable way, with voluntary participation (no personal data or identifiable responses were collected, even IP was not collected, or any cookie was set), consequently we did not initiate and obtain ethical approval for that.

The list of questions was designed in multiple rounds within the OSIRIS consortium which ensured that no harm or risk is posed to respondents.

Respondents, clicking on the survey link were navigated to an introduction page, where detailed information about the study was provided, including the link to the study protocol and the full list of questions, in advance, before starting the survey itself. In possession of this knowledge, participants had the chance either to access the survey by checking the “I agree to take part in the research” box, or to leave it without any consequence. Accordingly, respondents took part with electronically checked written consent.

In addition, the GDPR (Hungarian University of Agriculture and Life Sciences) office was consulted and informed about the nature and content of the study. They verbally informed us that GDPR is not a relevant issue in the case of this particular survey. The detailed consent page can be found in the OSF survey material in the OSF ( Gelsleichter et al., 2025).

### Data analysis

The survey comprised closed questions, supplemented by six open-ended questions at the end of the technical sections. These open-ended questions provided free text space for respondents to express their views about the given questions.

Closed questions were analyzed by descriptive statistics, prepared using Quarto ( Allaire et al., 2022) version 1.6.32 within RStudio ( Posit team, 2025) version 2025.05.0+496. The R ( R Core Team, 2024) used in the analysis can be found in ( Gelsleichter et al., 2025). Quarto made it possible to prepare the data analysis along with data visualization for the reporting materials, ensuring the reproducibility of the work.

Both quantitative and qualitative data were summarized using numbers and percentages. In the case of questions that asked respondents to select and rank options from predefined lists, a simple weighted scoring method was applied to evaluate ranking. Depending on the number of ranked items, rank 1 = 3 points, rank 2 = 2 points, and rank 3 = 1 point, as well as rank 1 = 5 points, rank 2 = 4 points, rank 3 = 3 points, rank 4 = 2 points, and rank 5 = 1 point, scoring was applied to convert rank percentages into a single composite score for each item.

Open-ended questions were partly categorized for a more conscious evaluation and interpretation of opinions. Categorization was made by MV and discussed, and consensus was reached between MV and YAG. Complete replies to open-ended questions can be seen in the respective OSF database ( Gelsleichter et al., 2025).

The questions were also analyzed using demographic group breaks. This analysis can be found in ( Gelsleichter et al., 2025). Countries were categorized into two groups, developed and developing, based on the UNDP classification (2025).

## Results

### Demographics of respondents

The survey was initiated by 254 respondents; 194 of them (76.4%) completed the demographic stage and were distributed across the six inhabited continents: *Respondents were mainly based in Europe 157 (80%), followed by Asia 18 (9.3%), North America 8 (4.1%), South America 7 (3.6%), Africa 2 (1%), and Oceania 1 (0.5%).* In total, 133 respondents completed the survey. The majority of the drops were immediately after the demographics; therefore, the study only considered 133 complete responses. Owing to the skipping mechanisms described in the survey design, the number of respondents varied across questions. Respondents from Europe constituted 109/133 (82%) of the survey. Regarding the scientific field of respondents ( Table 1), it is distributed across *Natural sciences* 32/133 (24%), *Medical* 28/133 (21%), *Agricultural* 27/133 (20.3%), *Engineering & Technology* 26/133 (19.5%), and *Social sciences & Humanities* 20/133 (15%). In line with the target population defined in the protocol, respondents were mostly *Researchers and Academics* (120 of 133 respondents, 90.2%). For the career stage ( Table 1), most (50 out of 133 respondents, 38%) were *Established researchers* based on the EURAXESS (2023) classification, followed by *First stage researcher II* (defined as: carry out research under supervision, graduate students; 30 out of 133, 22.6%). Considering the type of institutions, 67/133 (50.4%) responses were from *universities or higher education institutes*, 40/133 (30%) from *research institutes or research centers*, 8/133 (6%) from *non-profit organizations*, 7/133 (5.3%) from *government agencies or their departments*, 4/133 (3%) from *commercial entities*, 2/133 (1.5%) from *government operated commercial entities*, and 5/133 (3.8%) from *other* types of institutions (university clinic, university hospital, scientific journal, or did not want to disclose). The target population of the survey was not only computer scientists, but also all disciplines utilizing digital data collection and analysis tools. In line with this, as well as the well-balanced distribution of scientific fields among respondents, the survey represents various disciplines and provides a level of generalizability.

**Table 1.** Demographics, composed of *type of institution, field of research, interest, career stage.*

Category	Subcategory	Count
*Type of Institution*	*University or higher education institute*	73
	*Research institute (or research center)*	40
	*Non-profit organization*	7
	*Government agency or department*	6
	*Other ^1^*	4
	*Commercial entity*	2
	*Commercial where the government is a major stakeholder*	1
*Field of Research*	*Natural sciences*	32
	*Medical sciences*	28
	*Agricultural sciences*	27
	*Engineering and technology*	26
	*Social sciences and Humanities*	20
*Interest*	*Researchers and Academics*	120
	*Other ^2^*	6
	*Journal and Publication Professionals*	3
	*General Public*	2
	*Policy Maker and Governance*	2
*Career Stage*	*Established Researcher*	50
	*First Stage Researcher II*	30
	*Recognized Researcher*	18
	*Leading Researcher*	17
	*First Stage Researcher I*	9
	*Other ^3^*	9

Meaning of Others in the Type of Institution ^1^: *University Clinic, University hospital, Scientific journal,* and *I do not want to disclose it.* Each had one reply.

Meaning of Other in Interest ^2^: *Data sharing support, Data steward, IT support, Research reform advocate, Research Support services,* and *Statistical analysis.* Each had one reply.

Meaning of Other at Career Stage ^3^: *Retired researcher, now coordinating editor; Software developer (worked for researchers for 10 years), Semi-retired researcher, Data steward, Support staff, Research software engineer.* Each had one reply and *I do not want to disclose it* had three replies.

To illustrate the nature and focus of the respondent population, in the checkbox Question 3.3, which asks what *kind of research data you generate,* among several listed options, *Quantitative data* (numeric files, survey responses, geospatial data), *Omics data* (information generated by studies ending with -omics: genomics, proteomics, phenomics, etc.), *Imaging data* were selected mostly (91%, 21%, and 21% among 120 respondents, respectively).

### Open Science practices supporting computational reproducibility

**Figure 1.**
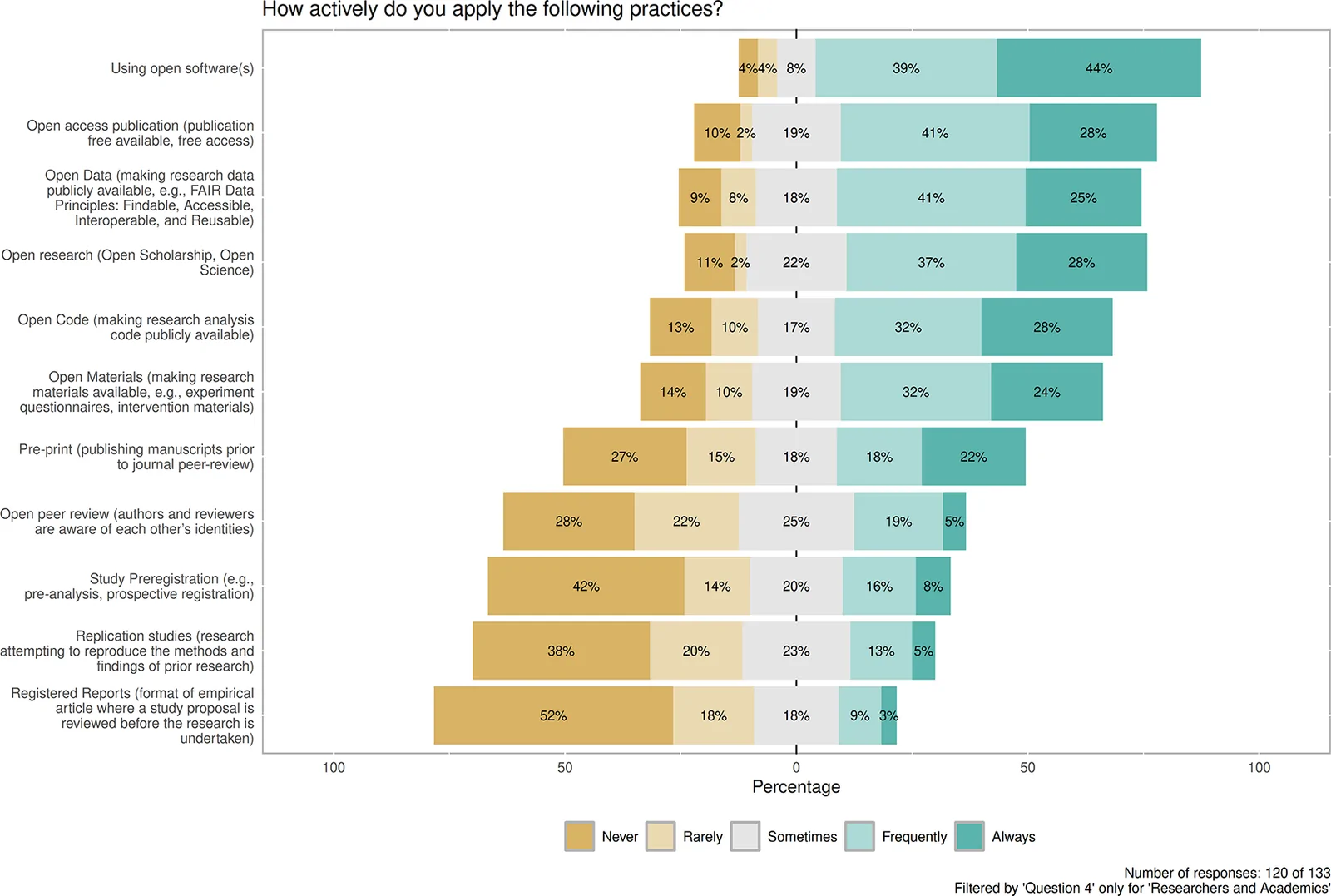
Application of open science practices. Contains the distribution between replies Never, Rarely, Sometimes, Frequently and Always in context of predefined practices. Number of responses: 120 of 133. Filtered by 'Question4' only for 'Researchers and Academics'.

Regarding the awareness and extent of use of practices supporting open science and computational reproducibility, a question focused on the *prevalence of various tools, methods and techniques, by listing 11 OS practices* to cover the available solutions as much as possible ( Figure 1). Replies shows that the *Use of open software* is a highly adopted practice (100/120, 83% of the respondents use them *Frequently* or *Always*). Followed by *Open access publication* with 83/120, 69% of *Frequently* and *Always* options suggests that it is also a widely known and used practice (meaning that “ *there are no financial, legal or technical barriers to accessing it*”), ( openaccess.nl, 2025).

*Open data*, *Open research (including open scholarship)*, *Open code and open materials* can be considered as less frequently applied practices among respondents with a range of 68–79 from 120, 56-66% of *Frequently* or *Always* replies.

On the less common side, *Registered reports*, *Replication of studies*, or *Study pre-registration* belong to less known and applied practices, where 62/120, 46/120, and 50/120 respondents (52, 38%, and 42%, respectively) *Never* used these practices. *Open peer review* was among the least common practices with 60/120 (50% of responses) when combining *Never* (34/120, 28%) and *Rarely* (26/120, 22%).

According to replies for question about *Sharing of data*, *Code* and *Research documentation* in case of work with public funding should be made accessible, according to respondents (96/133, 72%; 84/133, 63%; and 97/133, 73%, respectively). Although what is more notable here is the relatively high percentage of ‘neutral’ responses (37/133, 28%; 43/133, 32% and 31/133, 23%) which implies that there is still a great need to raise awareness and to develop incentive schemes.

**Figure 2.**
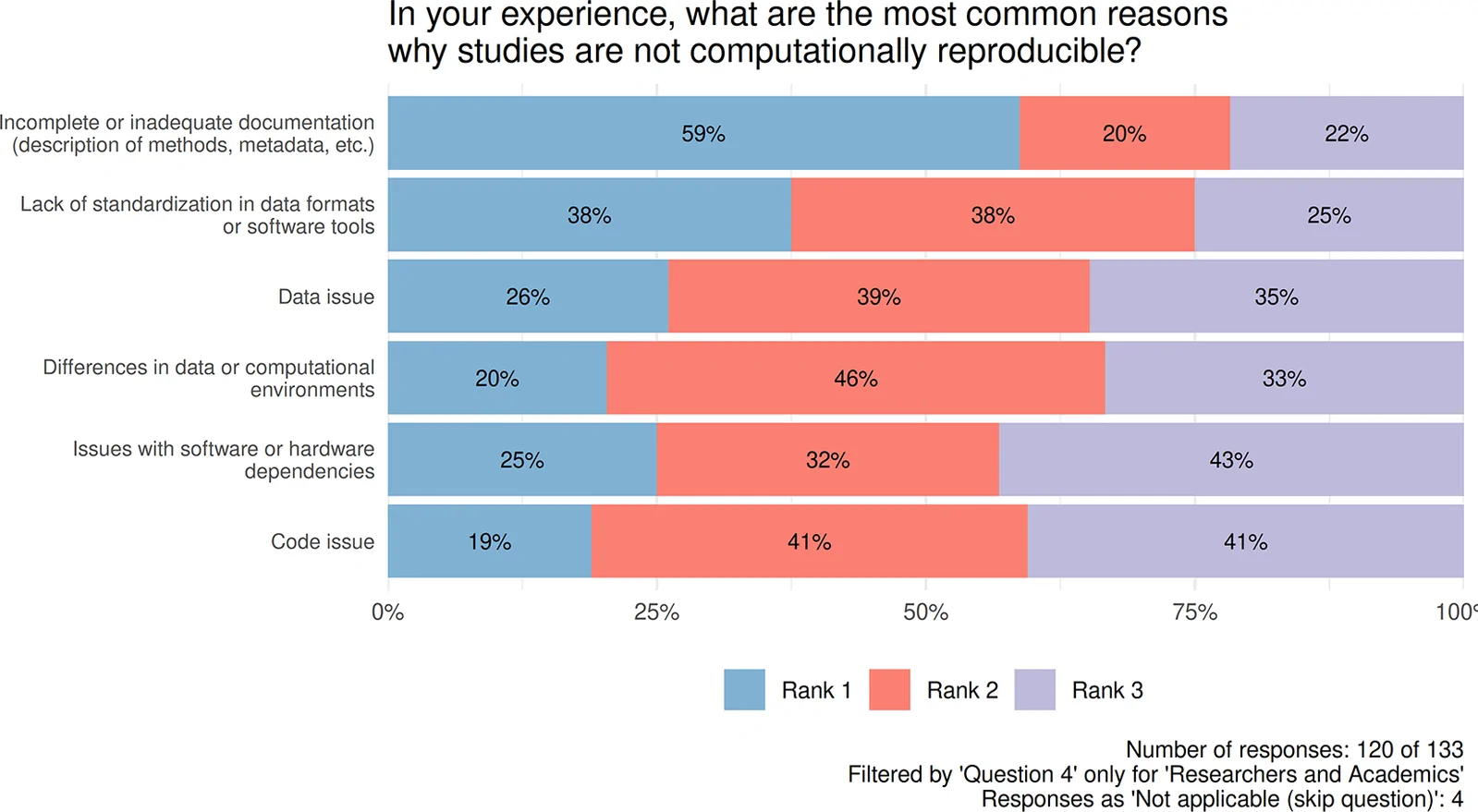
Ranked obstacles to computational reproducibility. Figure introduces the experiences about the most common reasons why studies are not reproducible. Respondents were asked to select and rank three items (Rank1, Rank2, Rank3) deemed the most important in their view. Number of responses: 120 of 133. Filtered by 'Question4' only for 'Researchers and Academics'.

In the question of revealing barriers to reproducibility ( Figure 2), respondents were asked to select and rank three items deemed the most important in their view. Based on the “Rank 1 = 3 points, Rank 2 = 2 points, Rank 3 = 1 point” conversion, *Incomplete or inadequate documentation* received a score of 287 (3 × 120 × 59% + 2 × 120 × 20% + 1 × 120 × 22%) at first place, followed by *Lack of standardization in data formats or software tools* with 258 points (3 × 120 × 38% + 2 × 120 × 38% + 1 × 120 × 25%), and *Data issue* with 229 points (3 × 120 × 26% + 2 × 120 × 39% + 1 × 120 × 35%).

In the degree of efforts made toward reproducing others’ work, 33/120 (27.5%) respondents *Never tried to reproduce a study*, while only 9/120 (7.5%) respondents reported *High degree of reproducibility*, compared to the 24/120 (20%) respondents with the opinion of *Low degree of reproducibility* or *Impossible to reproduce.* The number of replies with a neutral *Medium degree of reproducibility* was 53/120 (43.3%). Another question illustrates that according to experience, open data, open code or metadata, gathering *Never*, *Rarely*, and *Sometimes* appear in the publications studied by respondents with 70, 71%, and 86%, respectively.

**Figure 3.**
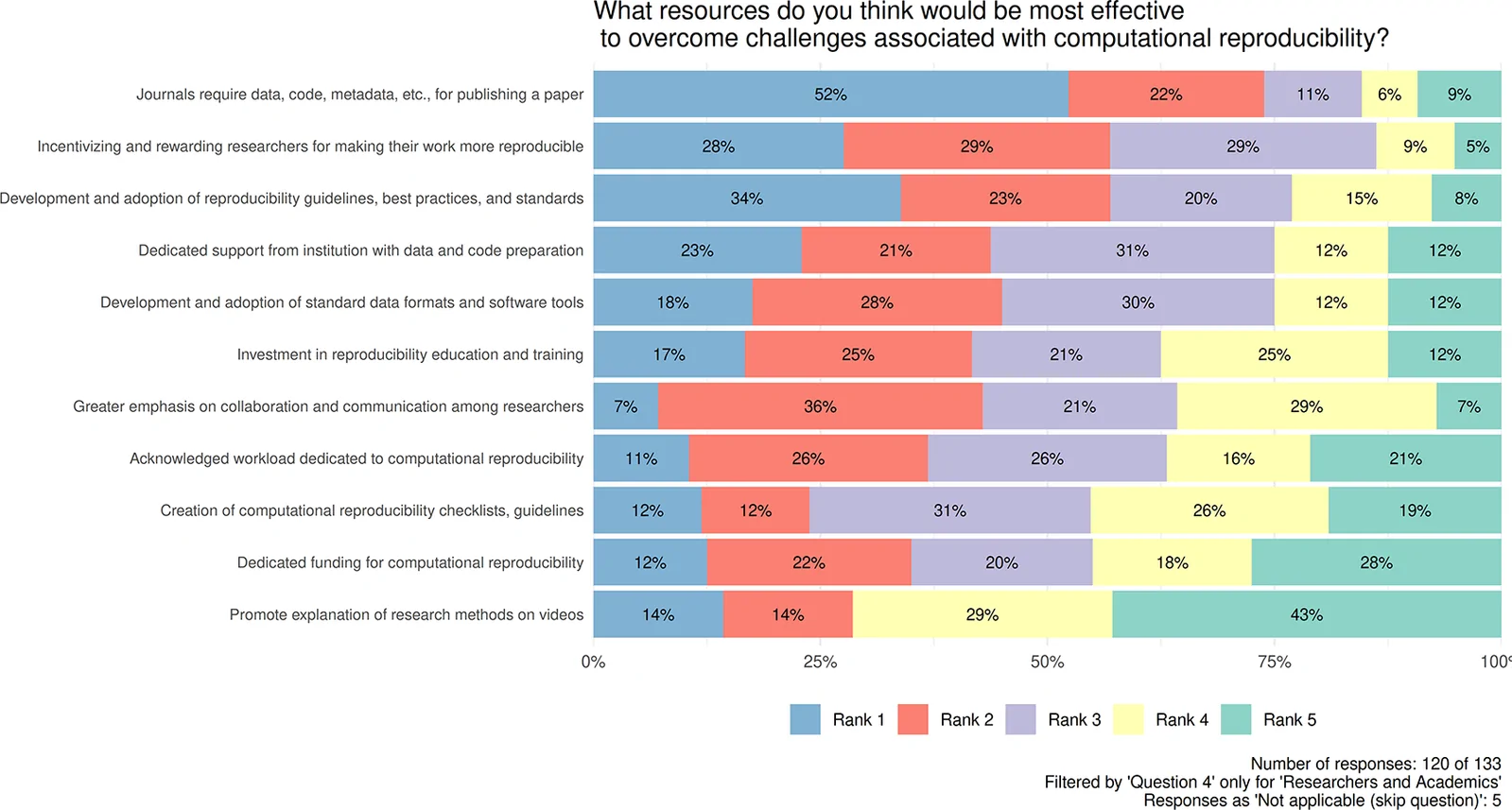
Strategies deemed important to overcome the challenges in computational reproducibility. Contains information about selection and ranking of up to five items (Rank1, Rank2, Rank3, Rank4, Rank5) that respondents deemed the most important from amongst the predefined list of 11. Number of responses: 120 of 133. Filtered by 'Question4' only for 'Researchers and Academics'.

Transparent and computationally reproducible research requires effort from researchers, accompanied by appropriate resources. A question to capture opinions about these resources was framed ( Figure 3), asking for the selection and ranking of up to five items that they deemed the most important from amongst the predefined list of 11 (note, *Not applicable* replies were removed from the analysis). Based on the responses of 120 academics/researchers and on the applied five-point scoring system, the most important driver is whether *Journals ask for the necessary data/code/metadata* (with scores of 482, 5 × 120 × 52% + 4 × 120 × 22% + 3 × 120 × 11% + 2 × 120 × 6% + 1 × 120 × 9%). This is followed by *Incentivizing and rewarding researchers for making their work more reproducible* (with a score of 439, 5 × 120 × 28% + 4 × 120 × 29% + 3 × 120 × 29% + 2 × 120 × 9% + 1 × 120 × 5%), followed by *Development and adoption of reproducibility guidelines, best practices, and standards* (with a score of 432, 5 × 120 × 34% + 4 × 120 × 23% + 3 × 120 × 20% + 2 × 120 × 15% + 1 × 120 × 8%). In the shared 4th place, *Development and adoption of standard data formats and software tools*, as well as *Dedicated support from institution with data and code preparation*, both with a score of 394 (5 × 120 × 18% + 4 × 120 × 28% + 3 × 120 × 30% + 2 × 120 × 12% + 1 × 120 × 12% and 5 × 120 × 23% + 4 × 120 × 21% + 3 × 120 × 31% + 2 × 120 × 12% + 1 × 120 × 12%, respectively). The 5th item of the list is *Investment in reproducibility education and training* with a score of 372 (5 × 120 × 17% + 4 × 120 × 25% + 3 × 120 × 21% + 2 × 120 × 25% + 1 × 120 × 12%).

**Figure 4.**
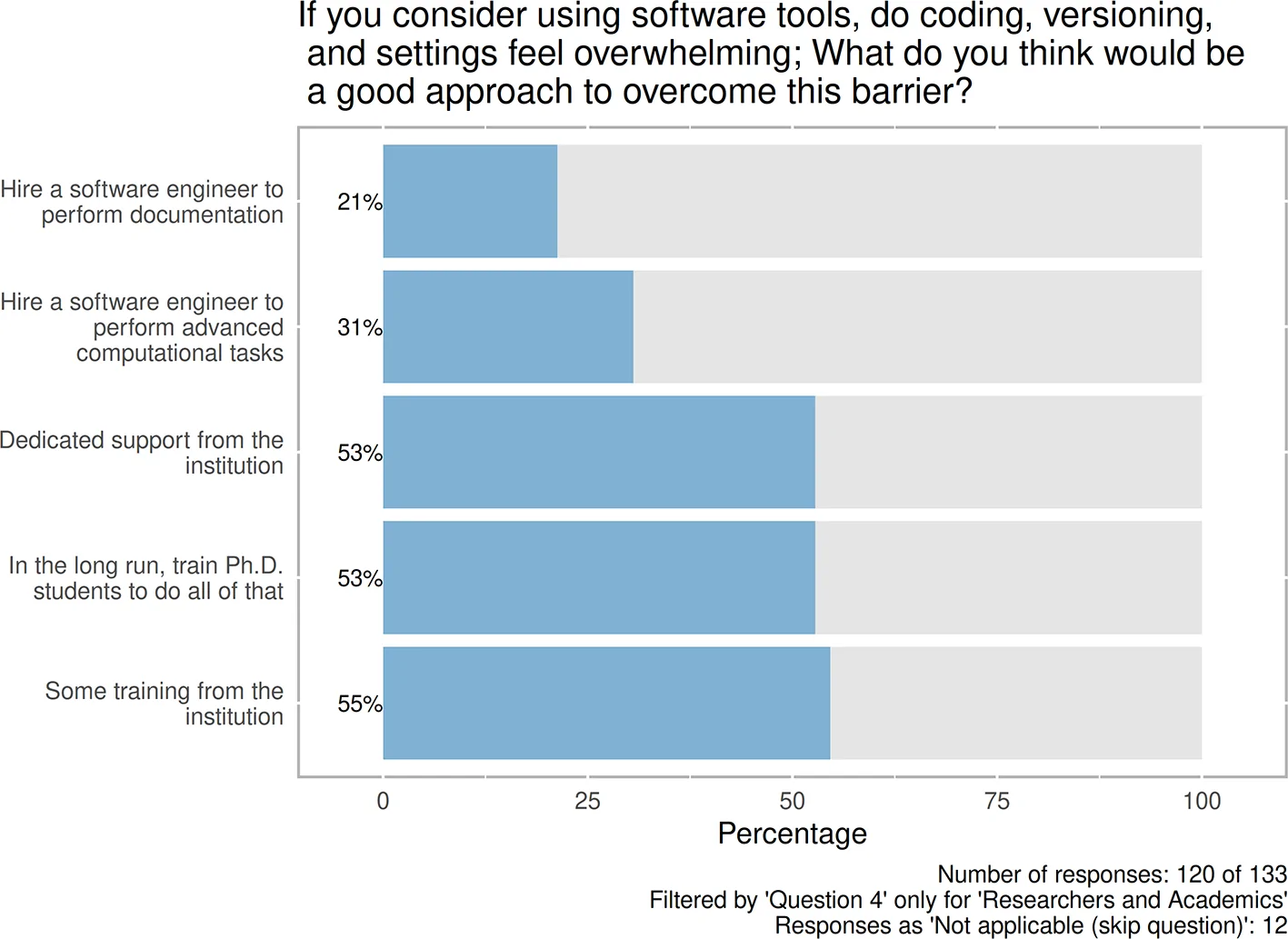
Actions to support computational work. Contains information about predefined and selected options that are considered important to overcome the barriers of computational reproducibility. Number of responses: 120 of 133. Filtered by 'Question4' only for 'Researchers and Academics'.

In addition, respondents were asked to check for the listed options that are considered important to overcome the barriers of computational reproducibility ( Figure 4). Among the five options, respondents highlighted the role of training in the first place, followed by the need for dedicated support, as well as training of PhD students in the long run (66/120, 55%; 64/120, 53%; and 64/120, 53%, respectively). In addition, the question offered an open-ended option, in which five opinions were shared. Four mentioned time, funding, and more conscious workflow management during the research process. One respondent expressed strong aspirations, namely “ *better criteria for job recruitment. If you cannot do this unsupported, you’re not supposed to be a researcher*”.

At the end of section 2, an open field was provided to allow respondents to share their insights about good practices that could enhance computational reproducibility. Twenty-one respondents provided detailed opinions categorized into five main topics (with some insights):

1. Incentives from journals/funding agencies/institutions (8 replies)
*“Journals should not only require, but also review the code and data for each submission.”*
2. Actual technological solutions (4 replies)
*“Using declarative deployment systems like Nix/Guix to limit issues related to dependencies and ease deployment.”*
3. Importance of behavior change, focusing on early career researchers (3 replies)
*“We also need to train people how to write good code and document things.”*
*“I also believe journals need more methodology review experts to evaluate thoroughness of reporting.”*
4. Trainings, guidelines, hackathons for improving skills (2 replies)
  “Repro hackhaton.”
5. Suggestions for dedicated positions (both at institutions and in journals) (2 replies)
*“Appoint Open-Science-Friendly engineers/researchers as referent within all research units to communicate with fellow researchers and convey good practices, with nationwide exchanges between referents within a national (possibly international) network.”*
*“Emphasize that it is a revolution, but it does not have to be all done right away => step-by-step process, project after project, improvements after improvements, mistakes are ok (even in codes and data). And not everybody can change how they do research at the same pace: list all that can be done and ask people what small changes they can do today? And what could they plan to do in the future?”*

Detailed responses can be found in the respective OSF database ( Gelsleichter et al., 2025).

### Sharing research data

Considering the equilibrated range of disciplines that participated in the survey, qualitative data (interviews, focus groups, field notes, images, audio, video, etc.) were selected by 22% of respondents. *If I do not produce data in my research* or *Not applicable* was, the following answers were skipped and respondents were taken to the next section: In the survey, 33/120 (27.5%) respondents were skipped, and 87/120 (72.5%) went through.

Respondents were asked to estimate the number of shared datasets over the past five years. As a result, more than half of the respondents (70/116, 60.3%) *Practice data sharing in their own work* (the responses are distributed as follows: 64 replies with 1-10 datasets, 3 replies with 14 datasets, 1 reply with 20 datasets and two replies with more than 30 datasets).

Responses show the popularity of *Data repositories* (Zenodo, Dryad, Mendeley data, Figshare) with 56/87 (64%), followed by *Supplementary materials* 45/87 (51%), and *Data papers* (Data in Brief, Scientific Data, etc.) with 32/87 (37%) among researchers who practice data sharing. As this question was a checkbox selection, participants were able to choose more than one option; therefore, each item had a hundred percent possibility. Other free box options also highlighted the option of GitHub, the OSF repository, and the project websites of funding bodies.

**Figure 5.**
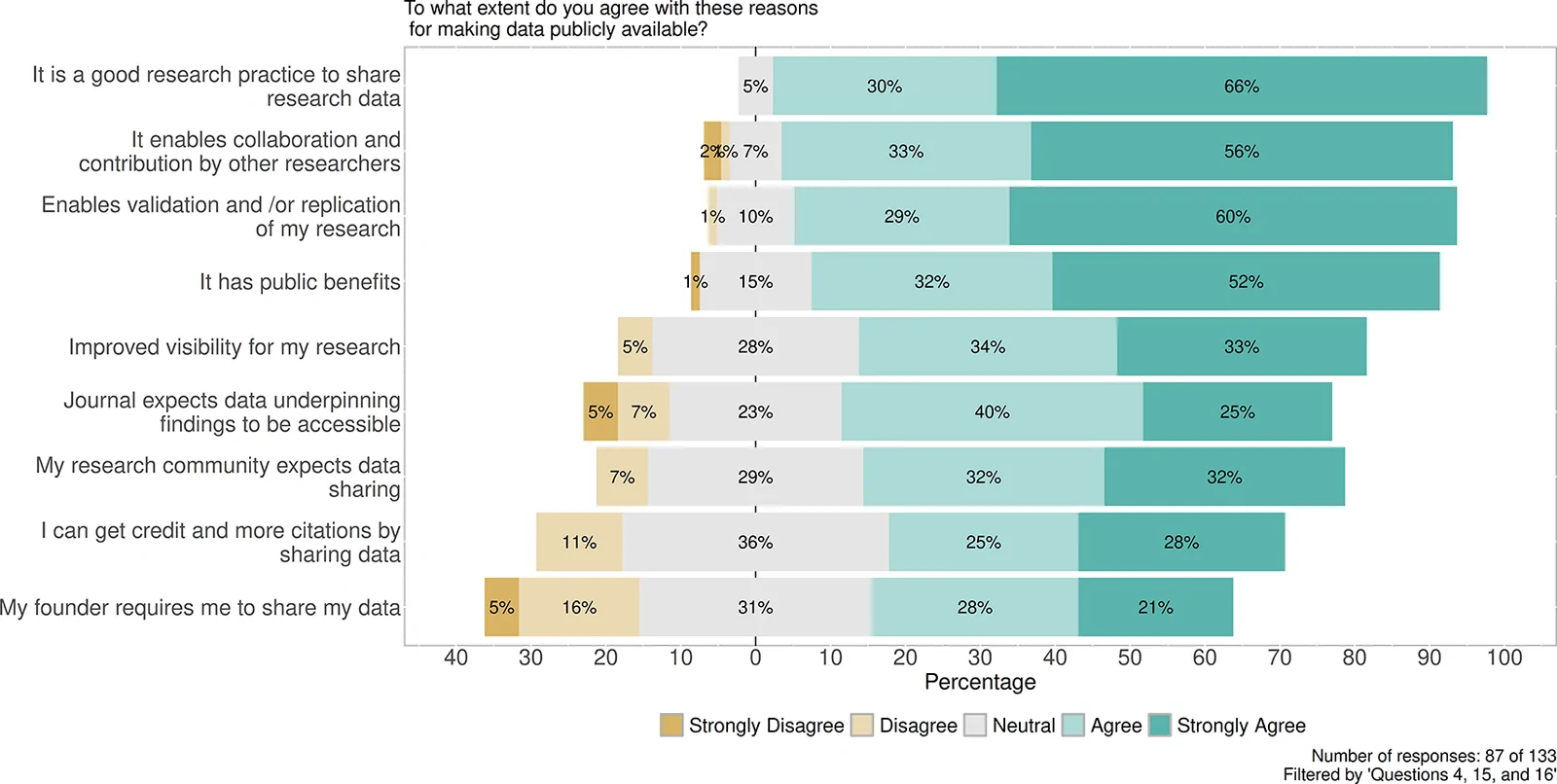
Reasons to make data publicly available. Contains the distribution between replies Strongly disagree, Disagree, Neutral, Agree and Strongly agree in context of the question why making data publicly available is important. Number of responses: 87 of 133. Filtered by 'Questions 4, 15 and 16'.

In response to the question ( Figure 5) *why making data publicly available is important*, different motivations were grouped by the level of agreement. *Because it is a good research practice* 84/87, (96%) respondents *Agree* (30%) and *Strongly Agree* (66%). At the same time, 77/87 (89%) respondents considered it important for *Enabling collaboration and contribution by other researchers*, *Agree* (33%) and *Strongly Agree* (56%), and close ratios for *Enabling validation and replication* (89%) when combining *Agree* (29%) and *Strongly Agree* (60%), and *Public benefits* (84%) when combining *Agree* (32%) and *Strongly Agree* (52%). Respondents agree or strongly agree with the options of *My funder requires* and *I can get credit and more citations* with 49/87 (56%) and 57/87 (65%), respectively.

**Figure 6.**
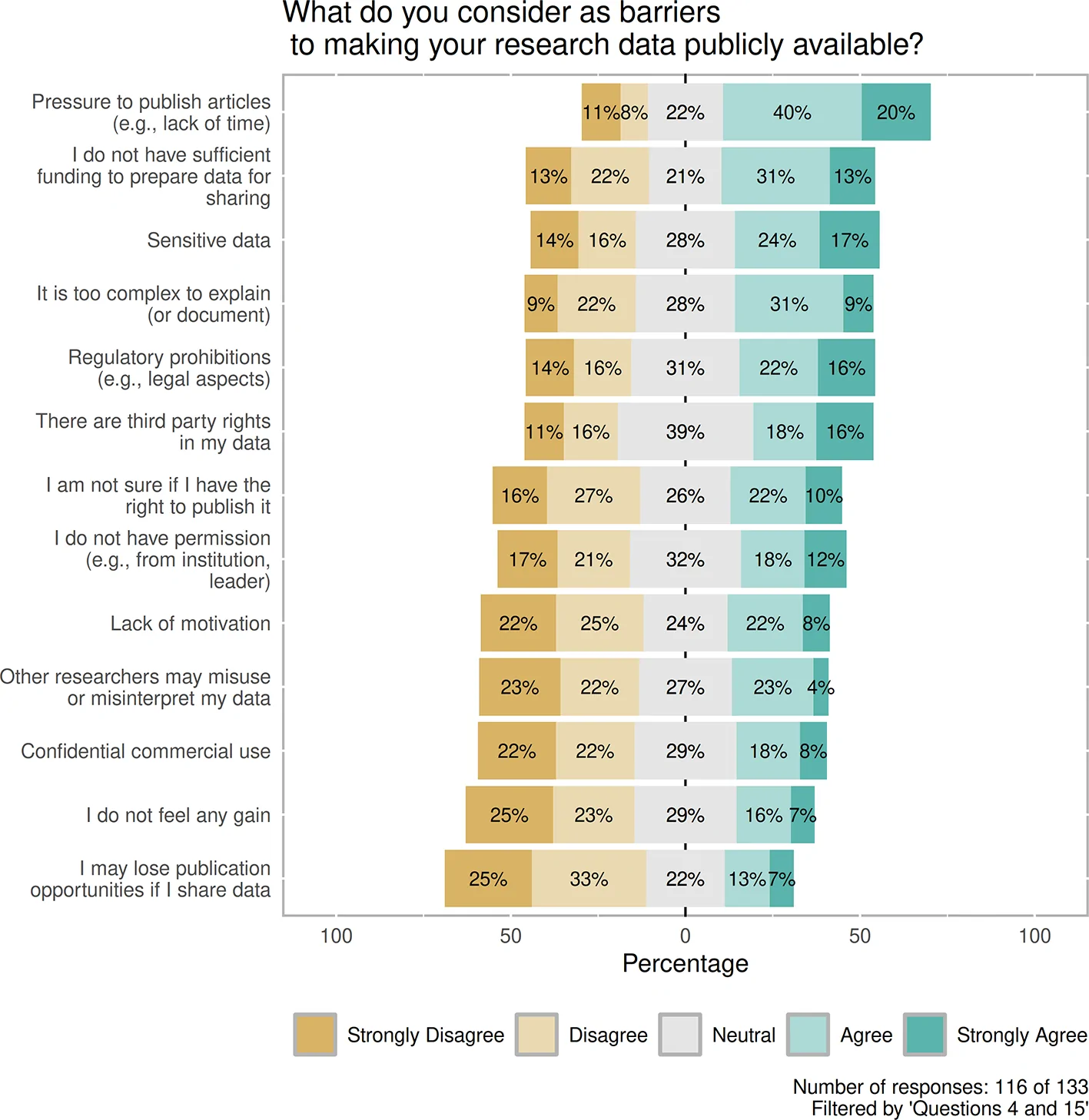
Barriers to making data publicly available. Contains the distribution between replies Strongly disagree, Disagree, Neutral, Agree and Strongly agree in context of predefined barriers. Number of responses: 116 of 133. Filtered by 'Questions 4 and 15'.

Considering the barriers ( Figure 6), respondents were asked to judge 13 predefined reasons that hindered data sharing. Here, the most common reason was the lack of time (i.e., the pressure to publish articles) (70/116, 60%), followed by the lack of sufficient funding that supports data sharing (51/116, 44%), and the sensitive characteristics of data were mentioned in third place (48/116, 41%) with *Agree* or *Strongly Agree.* Data complexity, uncertainty about rights to share, and lack of permissions can be considered moderate barriers, with a relatively high ratio of neutral responses, suggesting some level of perplexity. Factors of *Losing publication opportunities*, *Feeling of additional gain*, *Confidential commercial use* or *Lack of motivation* with a relatively high number of *Strongly Disagree* and *Disagree* options (67/116, 58%; 57/116, 48%; 51/116, 44%; and 52/116, 45%) show that these cannot be considered as major barriers.

**Figure 7.**
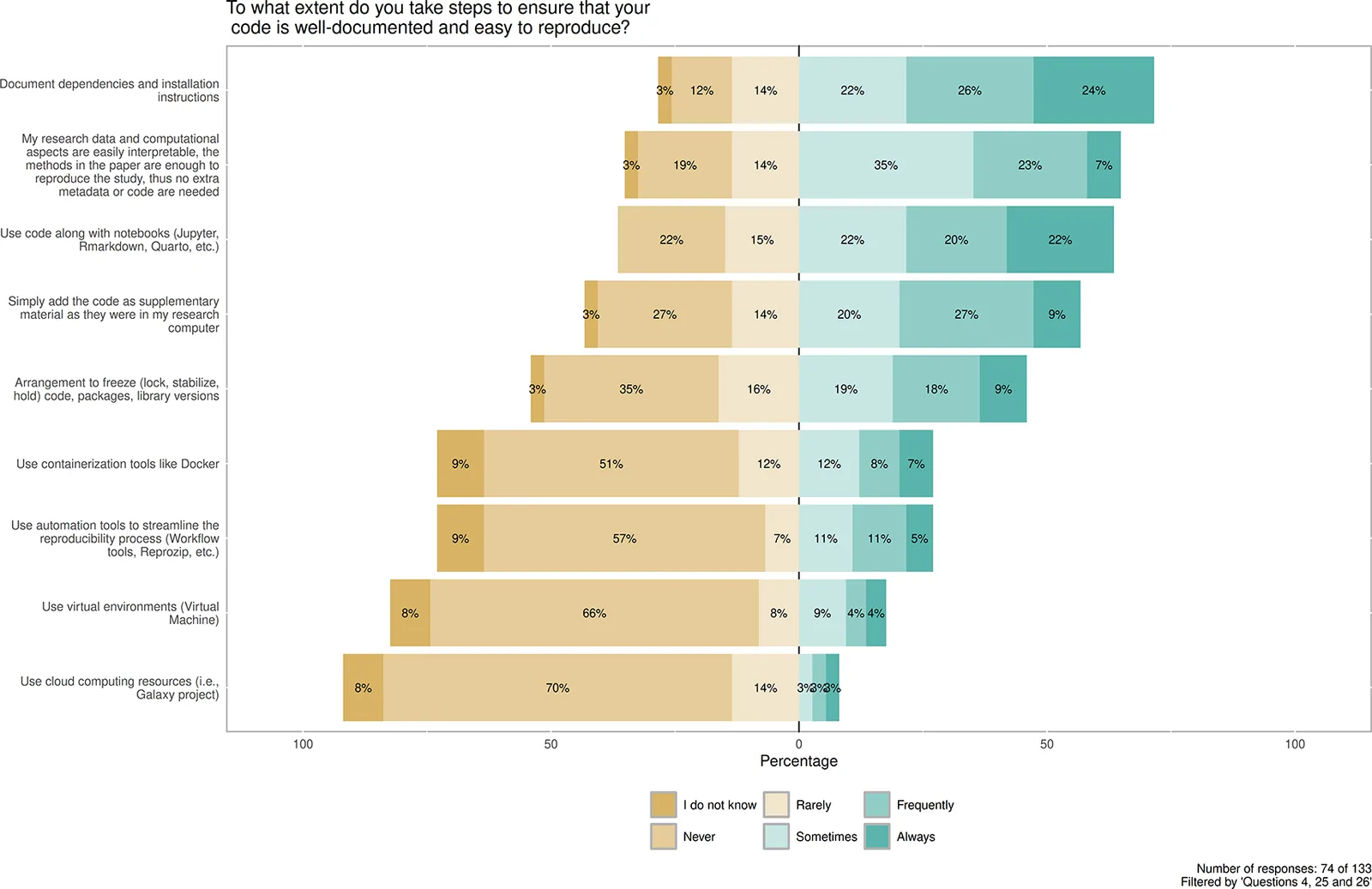
Actions made to enhance reproducibility. Contains the distribution between replies Do not know, Never, Rarely, Sometimes, Frequently and Always in context of predefined steps to making code well-documented and reproducible. Number of responses: 74 of 133. Filtered by 'Questions 4, 25 and 26'.

At the end of Section 3, 11 comments/opinions arrived, categorized into the following three topics, accompanied by meaningful replies (some examples are highlighted):

1. Regulations and requirements about data sharing (4 replies)
*“Simply it is not a requirement by education institutions. Otherwise lots of data would be available. And data sharing habit would start right away at university”.*
2. Technical issues (3 replies)
*“Make sure institutions/governments do not invent their own “data license” that is then hard to interpret.”*
*“Perfectionism (not wanting to publish data that is not processed/cleaned perfectly).”*
3. Behavior change (1 reply)
*“All publically funded research should require the data published.”*

Detailed responses can be found in the respective OSF database ( Gelsleichter et al., 2025).

### Data reuse

Regarding the reuse of existing data in Section 4, the most commonly mentioned purposes of utilization are for research validation, providing background or context to the given actual research, to reuse them in the development of their own methodology, and for teaching material. The relatively low response rate (30%) for replication and meta-analysis might also originate from definitional difficulties despite the explanatory pop-up messages of the survey. In the *Other* open box option, respondents also mentioned crowd-science projects, systematic reviews, or derivates data (e.g., maps from point data were actually referred). Only 6% of the 120 respondents stated that they had never used existing data. This is strong evidence for promoting data sharing, responses, and individual quotes to outline the actual state of data sharing and reuse and also implies the need for harmonized expectations that would most likely be highly supportive in terms of making data openly available (or at least accessible in line with the FAIR principles) as a norm in the research process.

### Insights about tools and code publishing

The aim of the respective question was to discover the extent of the use of different types of digitalized tools for data management and analysis. Considering the replies, R programming, and conventional spreadsheets (62/120, 52% of respondents stated that they use them *Always* or *Frequently* in both cases) are on the most popular side. In the middle range, Python programming language and various types of statistical software (SAS, SPSS, JASP, PSPP, GRETL, SOFA, KNIME, Scilab, etc.) were mentioned with 38/120, 32% and 36/120, 30%, respectively, considering *Always*+ *Frequently.* On the other hand, the least applied methods/tools are various, specific skill-requiring programming languages, programming platforms, and database management (68/120, 57%; 68/120, 57%; and 60/120, 50% never use them, respectively). Still, 28/120 respondents (23%) used analogical data collection. From the breakdown, less developed countries use around three times more paper and nearly twice the number of spreadsheets and statistical software, while more developed countries use more programming languages.

In addition to the eight predefined groups of tools, the next open-ended question aimed to identify other options. Here, seven additional responses arrived, highlighting mostly individual tools, for example, AI-based tools for preliminary analysis, various workflow tools, and application programming interfaces (in general, not further defined).

In a next question, respondents were asked to share their insights on whether the research code should also be evaluated in the peer review process by checking the most appropriate reply from ten predefined options. Twenty-one respondents selected *I do not know* (21/120, 18%), suggesting uncertainty about the topic.

Further, highly selected replies mostly state that it should be checked in various ways. Replies state that it should be performed only by a quick visual inspection (18), by machines (17), by a human staff of the journal (15), by a third-party operated cloud (13), a human researcher reviewer (13), or by a human from the journal staff, but only through a quick visual inspection (3). Compared to these numbers, only a few respondents (13) believed that the code should not be checked.

To discover ways of utilizing the research code, respondents were asked to check multiple predefined options that apply to their activities. Accordingly, 109/120 (91%) used it for data analysis; 103/120, 86% for visualization; 88/120, 73% for data cleaning; 84/120, 70% for the automation of the research process; 84/120, 70% for the organization of data and research work; 67/120, 56% for the collection of data; and 52/120, 43% for communicating the research work. In case *I do not use any code* 7/120, 6% were selected, further questions were skipped, and the survey was completed, ready to finish, and submitted.

To check the extent of sharing research codes, the next question aimed to gain insight into the number of shared research codes in the past five years, where, similar to data sharing activities, respondents were asked to type a number. Of the 133 people responded, 45 never shared codes, 55 shared 1-10 , and 13 shared more than 10 codes.

Mostly cited reasons to *making research code publicly available* are the followings:

• 70/74, 95% *Good research practice,* where *Agree* (26%) and *Strong Agree* (69%).
• 63/74, 91% *Enables validation and/or replication,* where *Agree* (26%) and *Strong Agree* (65%).
• 65/74, 88% *Enables collaboration and contribution by other researchers,* where: *Agree* (23%) and *Strong Agree* (65%).
• 64/74, 87% *Public benefits,* where *Agree* (30%) and *Strong Agree* (57%).

In the respective open-ended questions about code publishing, some of the challenges were highlighted, along with a claim for uncertainty about the question. A respondent raises attention to the point that code is useful only if it is “nice and tidy,” however, to make it such, it requires programming (coding) training, experience, and considerable time for cleaning and checking, commonly scarce resources in academia, unfortunately.

Considering code-sharing and publication practices in Question 5.8, replies were filtered by the number of shared codes in Questions 4.1, 5.4, and 5.5. Accordingly, 60/74 (81%) replies confirmed that most of the respondents who shared code *Use comment options to explain the code* parts and document all steps in the script. A similar ratio *Use README file for explanation* 59/74 (79%) or code publishing platforms (GitLab, Bitbucket, GitHub, etc.) to make the code publicly available 52/74 (70%). Version control was underrepresented by 41/74 (56%), despite its importance in code development. Specific code publishing platforms (like MethodsX, SoftwareX, etc.), as well as community or educational networks, are not typically used (5, 4%, and 3%, respectively) among respondents. Under *Other options* free text box, OSF, R Markdown, and Software Heritage Archive were also mentioned as applied solutions.

Continuing with the well-documented and easy-to-reproduce characteristics of shared codes ( Figure 7), respondents were asked to decide on a Likert scale (using *Always, Frequently, Sometimes, Rarely, Never*, plus *I do not know*) about efforts. On the most applied side, *Documentation of dependencies and installation instructions* (53/74, 72%) for *Always, Frequently* and *Sometimes* combined), and *Using code along with notebooks* (48/74, 65%) for *Always, Frequently* and *Sometimes* combined) are marked as applied practices. Respondents who shared code frequently never used cloud computing resources (52/74, 70%), virtual environments (49/74, 66%), automation tools (such as workflow tools or Reprozip ( Chirigati et al., 2016)) (42/74, 57%), or containerization tools (such as Docker ( Merkel, 2014)) (38/74, 51%).

For the respective open-ended questions about additional *aspects of code documentation*, seven replies were received. One part mentioned utilized tools (e.g., Guix, R script), and another part of replies highlighted the challenges of computational work:

• *“I usually only write R scripts and comment them, I feel I should do more with version control but I do not…”*
• *“I am aware of most of these tools and how it could/should be done (with containers etc.), but I never ventured so far, because my studies are not so general that I think anyone would touch it.”*
• *“Often projects have a combination of pipelines and notebooks this makes it very messy to share and takes time to organise but it is possible.”*

**Figure 8.**
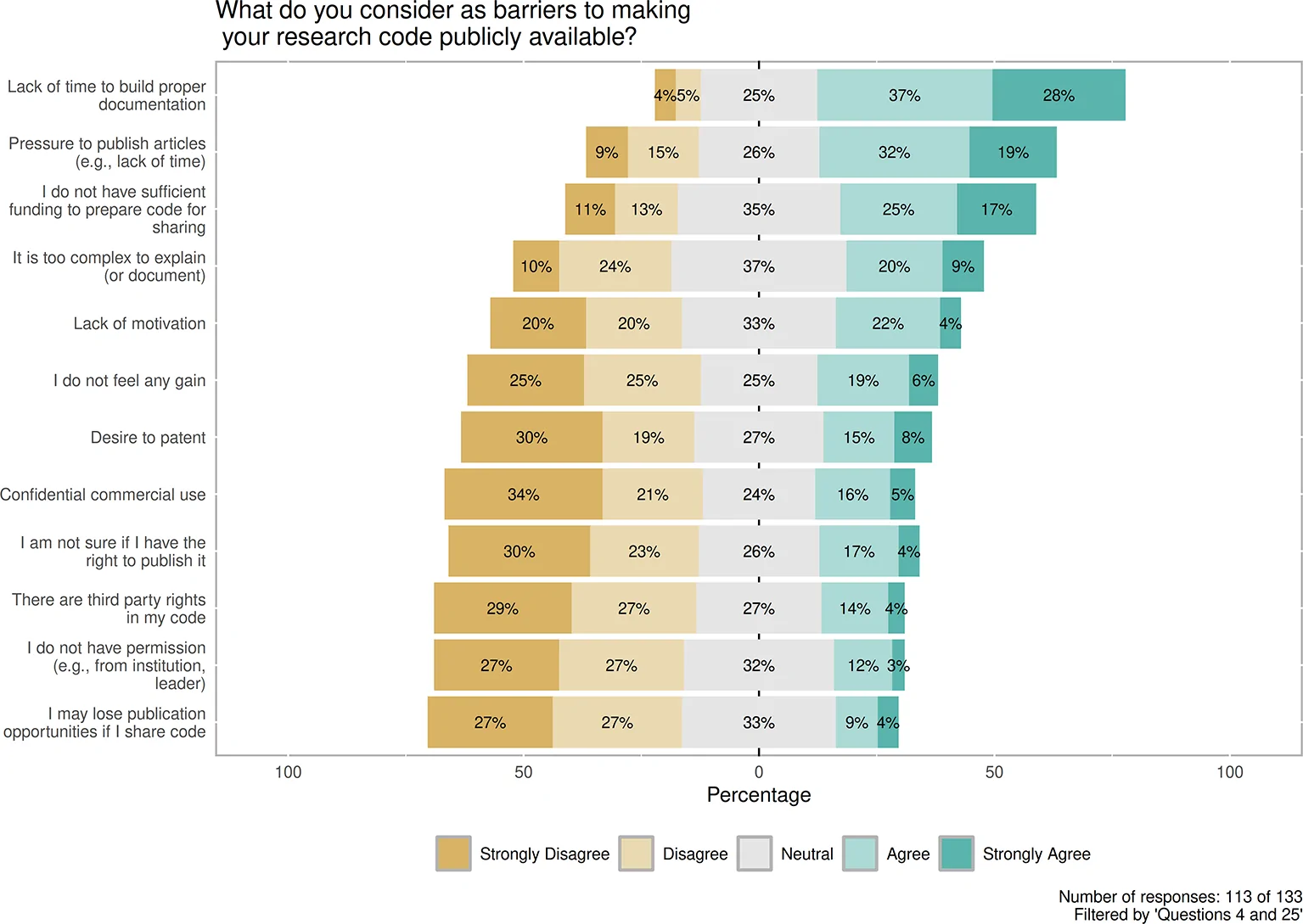
Barriers to making code publicly available. Contains the distribution between replies Strongly disagree, Disagree, Neutral, Agree and Strongly agree in context of predefined barriers to making code publicly available. Number of responses: 113 of 133. Filtered by 'Questions 4 and 25'.

Although respondents were aware of *Good research practices*, these replies were projected to the subsequent question ( Figure 8) about *barriers to making research code publicly available.* Fully in line with these highlighted free-text opinions, among predefined barriers: the *Lack of time to build proper documentation* 73/113, 65% ( *Agree* (37%) and *Strong Agree* (28%)); the *Pressure to publish* 58/113, 51% ( *Agree* (32%) and *Strong Agree* (19%)); and *insufficient funding to prepare code for sharing* 47/113, 42% ( *Agree* (25%) and *Strong Agree* (17%)) are the most commonly mentioned reasons. The following reasons are not considered barriers, as respondents voted with *Disagree* and *Strongly disagree*: *I may lose publication opportunities if I share code* and *I do not have permission* both with 61/113, 54% ( *Disagree* (27%) and *Strongly disagree* (27%)).

The open-ended question of the section provided space to add aspects of the limitations of code-sharing and publishing. In this question, six open-text replies arrived. The following three highlighted opinions raise points about the previously mentioned barriers:

• *“There are activities that are more highly valued that i do instead (publishing).”*
• *“I have to actively fight my PhD advisor to package my code with guix and really document the deps down to the kernel, because he reckons a requirements.txt is enough and I’m wasting the project’s time.”*
• *“To me, publishing the code is a way to document the scientific process. Unfortunately, some people expect the code to be reusable without any effort (to analyze another dataset for instance), which is not the purpose of publishing the code (publishing a package/software is a totally different process). This misunderstanding often leads to uninteresting email debugging discussions, which could discourage a “full publication” strategy of the research code.”*

### Insights about code reuse

Regarding the reuse of the research code, respondents were asked to check all the options that apply to their practices. Accordingly, a high proportion of respondents utilized the code to improve their own research code or to learn new coding strategies (86/113, 76% and 75/113, 66%, respectively). Research validation, replication, or utilization as a teaching material was in the range of 46-51 respondents out of 113, 41-45%, while 15/113 (13%) did not use existing research codes. Considering the credibility of reused research codes, researchers deem open accessibility and well-documented codes crucial.

Regarding the aspects of using existing code, the most important factor is well-established documentation, with 84%, followed by open-access code (83%), and 56% are concerned about acquiring code from a reputable source. Clearly defined rights to use, reference for code in research papers, immediate access, and the possibility to cite are less relevant, with 43, 50, 54, and 45%, respectively.

## Discussion

Discussion is organized following the structure of research questions.

### Perceptions about practices that support computational reproducibility

Key message: Researchers associate reproducible practices with transparency, collaboration, and public benefit. While awareness of the importance of reproducibility is widespread, there is still a gap between recognition and consistent implementation, with many researchers being cautious about sharing before publication.

When research is publicly funded, more than 60% of the respondents agree to share codes, data, and documentation. However, nearly 20% of leading researchers disagree with sharing documentation, mentioning time and effort as a burden.

Considering the reasons why making data publicly available is important, responses highlighted the following reasons: Because it is a good research practice was considered by 84/87 (96%) of respondents with Agree 26/87 (30%) and Strongly Agree 57/87 (66%). In fact, social desirability ( Fisher, 1993) is difficult to filter here. However, it is more meaningful that, in second place, 77/87 (89%) (Agree 29/87, 33% and Strongly Agree 49/87, 56%) of participants responded to *Enabling collaboration and contribution by other researchers*, together with similar ratios for *Enabling validation and replication and public benefits* 77/87 (89%) (Agree 25/87, 29% and Strongly Agree 52/87, 60%). Comparing the reasons for making the research code publicly available, a similar trend can be observed. Again, social desirability can appear; however, replies confirm that researchers are aware of the need to share data and codes.

The results highlight a gap between awareness and consistent implementation of reproducible practices. As pointed out by ( Stodden, 2010), researchers avoid revealing work before publication as a window of protection while ideas are still in development. In the same perspective ( Tenopir et al., 2011), found that only 30.5% of the scientists agreed to share data before publication.

On the other hand, the least indicated reasons for researchers sharing their code are the founder requirements, as well as obtaining more credit and citations. This shows a certain level of awareness, as good research practice, validation, and collaboration opportunities precede obligations among respondents. However, considering resources ( Figure 3), respondents ranked journal requirements first as they would create equal requirements and established expectations, thereby promoting reproducibility.

According to the replies, when the study received public funding, more than 60% of participants agreed to share Code, Data and Documentation. A similar point was noted by ( Tenopir et al., 2011), publicly funded research must be public property. In contrast, it is interesting to point out that, in this research, nearly 20% of the leading researchers disagreed with sharing research documentation when looking at the demographic breakdown. This might be because the concept of documentation can be cumbersome and time-consuming. Accordingly, behavioral change and education in the context of OS practices are still strongly needed.

### How code and data are shared during publication

Key message: Open-source software is considered the cornerstone of reproducibility. A large majority of the respondents (83%) reported frequent or always using open-source tools. Open-access publication is one of the most widely adopted practices (69%), although open peer review is less common and often not under the direct control of researchers.

Open-source software is a key element in many tools and services, and it inherently supports reproducibility. As (NI4OS, 2023) states, “the software-based services and infrastructure of OS are so important that it is safe to say that OS would not exist today without software, and, for a large part of that claim, without free and open-source software.”

The results show that the use of open software was adopted by 100/120, 83% (Frequently + Always) ( Figure 1) of respondents, demonstrating that the participants aligned OS practices with free software.

Scientific communication is moving into a new stage defined by transparency and reproducibility ( Stodden, 2010). The Association of Scientific, Technical & Medical Publishers (STM) found that Open Access (gold and green) publications increased from 20% to 43% between 2013 and 2023 ( STM, 2025), taking into account articles, reviews, and conference papers. In this survey, open access publication was in the second position among the most commonly used practices with 83/120, 69% (Frequently + Always in Question 2.1, Figure 1). However, Open peer review is a less common practice, mostly outside the researcher’s control. The results also highlight that other actors in the research ecosystem, such as journal publishers, play an essential role in disseminating good practices.

### Obstacles impeding computational reproducibility

Key message: Incomplete or inadequate documentation was the most frequently identified obstacle and was consistently ranked first by respondents. The lack of standardized practices across laboratories and research groups creates additional difficulties. Behind these shortcomings, the time and labor required to prepare materials for sharing, lack of time, and pressure to publish are mentioned. Cultural barriers such as reluctance to share unfinished work also contribute to inconsistent reproducibility. Structural obstacles include limited incentives, fragmented requirements, and lack of institutional support.

Incomplete or inadequate documentation is the top-ranked reason why the studies are not computationally reproducible. A similar aspect, lack of documentation, was found by ( Reinecke et al., 2022). On this topic, there is a trade-off between sharing and reproducibility. Although researchers do not prepare proper documentation, they are not willing to share data, code, etc., for several reasons; consequently, less material is available for computational reproducibility. On the other hand, sharing without basic organization and documentation is unlocking this ‘first’ barrier but not smoothing out the second one, still leaving a gap in this stage. Balance might be achieved through cultural change through training and coordinated requirements. To overcome the reproducibility barriers, respondents ranked first in Journals require data, code, metadata, etc. (with a calculated total score of 482, Figure 3), followed by incentivizing and rewarding researchers to make their work more reproducible (with a score of 439, Figure 3).

Journal requirements for code sharing have increasingly appeared in the past, particularly in fields where computational methods are central to research. Having studied the actual policies for some of the most acknowledged publication platforms (without claiming to be exhaustive), the code-sharing requirements are as follows:

• The authors are encouraged to make all the custom codes used in their research publicly available. Code availability is required for some journals in *Nature* Portfolio (such as *Nature Methods*), code availability is *required* (Nature Journal).
• Strongly encourages code sharing, and in many cases, peer review and publication are required (Science).
• Requires that all data and codes needed to replicate the results are made available without restriction at the time of publication (PlosOne).
• Strong emphasis on reproducibility. The code should be shared for all results derived from the computational analyses (eLife).

However, these incentives on the journals’ side ideally should be accompanied by actions on the institutional side to create a more supportive research environment in the long run.

The inconsistent standardization within single laboratories or research groups, along with the lack of measures such as systematic code commenting and documentation practices, are obstacles to computational reproducibility ( AlNoamany & Borghi, 2018). Considering that a 5-year-old paper ( Liu & Salganik, 2019) highlights the same issues against computational reproducibility, it is an alarming sign that there is still much to do toward progress in this field. ( Hocquet & Wieber, 2021) draw attention also to the “naïve expectancy of total computational reproducibility” as well as to the fact of epistemic issues in the actual practice. Accordingly, it is still an actual task to provide optionally specified guidance materials and train researchers about tools to standardize computing environments (e.g., dockerization and standardized OS platforms) in everyday research practice. The ranked items also highlighted the need for stronger collaboration between stakeholders (researchers, their institutions, and publishers) to create a more supportive environment.

As barriers to making research codes publicly available, respondents placed the first lack of time, followed by pressure to publish. In the same direction ( Stodden, 2010), found that the largest barriers to sharing code are the time to clean up and document it for release, followed by issues in the code by other users.

The responses and individual opinions highlight the need for harmonized incentives and awareness raising in all stages of the researchers’ life cycle and point out that publishing data and code in a computationally reproducible manner requires additional efforts from both the provider and the utilizer.

### Replication practices and success rates

Key message: Almost one-third of the respondents reported that they had never tried to reproduce another study. Based on the identified reason, time and labor constraints limit researchers’ ability to invest in replication. When replication is attempted, researchers often find that open data (70%), open codes (71%), and metadata (86%) are missing or incomplete. Respondents suggested that replication would be more successful if it was incentivized and better integrated into everyday research practices.

The survey also aimed to reveal the degree of effort made to reproduce others’ work. Importantly, 33/120 (27.5%) respondents had never attempted to reproduce a study.

In comparison with “59% of all participants never ran somebody else’s model to reproduce their results” from ( Reinecke et al., 2022), although their research focused on earth sciences researchers, instead of a broad target group like the present study. Consequently, these numbers suggest another intervention point to encourage reproducibility studies. However, considering time and labor constraints, it should be done and incentivized in a reasonable manner, linked strongly to actual research work. According to respondents’ experiences, Open data (84/120, 70%), Open code (85/120, 71%), or Metadata (103/120, 86%) are Never, Rarely, or only Sometimes available in the publications they are reading.

## Study limitations and recommendations

Given the method of distribution, we did not have information about either the reached population or response rate. However, the demographics of the population who completed the survey are described in detail in the Demographics section.

The likely low response rate due to the survey’s broad distribution limits its representativeness. Accordingly, the findings may not reflect the broader research community, especially because demographics (mainly region) of respondents cannot be considered representative, as most replies arrived from Europe (109/133, 82%). Despite these limitations, the study aligned findings with similar previous studies (e.g. Liu & Salganik, 2019; Reinecke et al., 2022), demonstrating consistency in identified trends and challenges in open science practices. Nevertheless, it is well-balanced in terms of disciplinary breakdown, and despite limitations, it provides an up-to-date snapshot about the most and least common open science, data, and code sharing practices, key barriers, and highlights the need for a systemic cultural shift within the research ecosystem.

As a recommendation, a lower number of questions may lead to more direct questions and focused results.

## Conclusions

Digital tools play a crucial role in reproducible research by enabling standardization and automation; supporting data provenance and metadata tracking to ensure traceability and integrity; and facilitating transparent and shareable reporting. The survey revealed that barriers to computational reproducibility remained largely unchanged over the past six years ago ( Liu & Salganik, 2019) and three years ago ( Reinecke et al., 2022) pointed out the same challenges regarding data and code sharing. Issues such as inadequate documentation, incompatible computing environments, and unresolved software dependencies, as well as a lack of time, continue to hinder progress. This should be a key consideration and a focus on current and future metascience projects.

Practices to support computational reproducibility, such as the use of open-source software and open access publishing, are now well established among researchers, demonstrating a strong foundation for open science principles. However, this widespread awareness has not yet translated into the consistent implementation of more demanding reproducibility practices, such as study (pre) registration, replication efforts, or open peer review, which remain significantly underutilized. Moreover, although most respondents agreed that data and code sharing are vital for scientific integrity and collaboration, actual sharing practices lag behind, with a large portion of researchers reporting little or no data or code publications in recent years.

These persistent challenges underscore the importance of providing effective technical support for researchers in the form of standardized tools, training, and methodological guidance to help overcome practical obstacles and to utilize computational methods more routinely. Training initiatives were identified as crucial for embedding good practices early in the research lifecycle. Embedding data management and data analysis into PhD, or even Bachelor’s (BS) and Master’s (MS) programs, is crucial across disciplines, as most of them increasingly rely on collecting, managing, and interpreting data.

A recurrent theme throughout the survey was the need for structural incentives and institutional support. Researchers claim that making work reproducible requires time, resources, and expertise; however, these efforts are rarely rewarded in outdated, conventional academic evaluation systems. Respondents also emphasized the role of journals, funding agencies, and institutions in promoting and rewarding open and reproducible research. These opinions highlight the need for the wider dissemination of new evaluation systems (DORA: Cagan, 2013; CoARA, 2022) that have not yet been widely applied.

In conclusion, although awareness of open science and reproducibility is high, widespread and consistent applications are still lacking. Addressing this gap requires coordinated efforts to remove technical barriers, redesign incentive structures, and create a culture supporting transparency and collaboration. The insights from this survey suggest that meaningful progress will depend not only on individual effort but also on systemic change across the research ecosystem.

## Software availability statement

For the analysis, Quarto ( Allaire et al., 2022) version 1.6.32 within RStudio ( Posit team, 2025) version 2025.05.0+496. R ( R Core Team, 2024) was used, that is an open-source scientific and technical publishing system, available at https://quarto.org/.

## Data Availability

Open Science Framework (OSF): 3.1. Computational reproducibility checks, Folder “Survey raw data and analysis”, https://doi.org/10.17605/OSF.IO/6YSRH, Gelsleichter et al., 2025. Folder contains the following files:
•
Survey_documents_from_LimeSurvey.zip – the complete set of materials implemented in the LimeSurvey online platform ○ Folder “form_and_questions” contains the original survey materials, exported from the LimeSurvey platform, including a README - Computational reproducibility status survey.txt that supports overview of underlying files. ○ Folder “survey_responses” contains the raw anonymous responses, exported to several formats allowed by LimeSurvey platform, along with a README.txt •
Survey_responses_analysis.zip contains the analysis of survey responses, organizing the respective files in the following folders: ○ Folder “input”: contains the raw input files and other annexes used for the analysis. ○ Folder “script”: contains the complete analysis script to support reproducibility, as well as the result files ○ Readme.txt Open Science Framework (OSF): 3.1. Computational reproducibility checks, Folder “Protocol_survey”, https://doi.org/10.17605/OSF.IO/6YSRH, Gelsleichter et al., 2024. OSIRIS_survey_protocol.pdf contains the protocol of the study, the informed consent page of the survey, as well as the survey itself with the complete list of questions. Open Science Framework (OSF): 3.1. Computational reproducibility checks, Folder “CHERRIES checklist”, Checklist for ‘Survey about Barriers and Solutions for Enhancing Computational Reproducibility in Scientific Research’, https://doi.org/10.17605/OSF.IO/6YSRH, Gelsleichter et al., 2024. Data are available under the terms of the Creative Commons Attribution 4.0 International license (CC-BY 4.0).
