# Exploratory Insights into Barriers and Open Practices for Computational Reproducibility in Scientific Research

**DOI:** 10.12688/f1000research.172013.3

**Published:** 2026-08-11

**Authors:** Yuri Andrei Gelsleichter, Rita Banzi, Florian Naudet, Constant Vinatier, István Kertész, Monika Varga

**Affiliations:** 1 Department of Soil Science, Institute of Environmental Sciences, Hungarian University of Agriculture and Life Sciences, Gödöllő, 2100, Hungary; 2 Laboratory for Health Regulatory Policies, Mario Negri Institute for Pharmacological Research IRCCS, Milan, Italy; 3 CHU Rennes, Inserm, Irset (Institut de recherche en santé, environnement et travail)-UMR_S 1085, CIC 1414 (Centre of Clinical Investigation of Rennes), University of Rennes, Rennes, France; 4 Institut Universitaire de France, Paris, France; 5 Institute of Food Science and Technology, Hungarian University of Agriculture and Life Sciences, Budapest, 1118, Hungary; 6 Institute of Animal Sciences, Hungarian University of Agriculture and Life Sciences, Kaposvar, 7400, Hungary

**Keywords:** Open science; Open Data; Open Code; Research transparency; Science integrity; Open Survey; Reproducibility

## Abstract

**Background:**

Rapid adoption of digital technologies across research disciplines underlines the need for accessible and reusable computational data and code.

**Methods:**

An anonymous, multidisciplinary survey examined researchers’ perceptions, needs, barriers, and self-reported practices concerning open science, data and code publishing and reuse.

**Results:**

Of 254 respondents who initiated the survey, 133 completed it, mostly from Europe. Registered reports, replication studies and pre-registration were among the least frequently reported practices (52%, 38% and 42%, reported as *Never applied*), while open software and OA publishing demonstrated widespread adoption (83% and 69%) of the respondents, respectively. The main perceived barriers to data sharing were *lack of time* (60%) and *insufficient funding* (44%). For code sharing, they were *lack of time to prepare documentation* (65%), *publication pressure* (51%), and *insufficient funding* (42%). Journal requirements (score: 482) and institutional incentives and rewards (score: 439) were the highest-ranked supporting measures. 28% of respondents indicated that they never tried to reproduce a study, and when replication was attempted, researchers often found that open data (70%), open code (71%), and metadata (86%) were never, rarely, or only sometimes available in the publications they read.. Open-ended responses emphasized training, career-stage guidelines, and basic programming skills.

**Conclusions:**

As survey was disseminated through open-science channels using volunteer sampling, no response rate could be calculated, and the sample was self-selected toward researchers already engaged with reproducibility. Findings should therefore not be generalized to the wider research community. Within this group, the central finding is a gap between awareness and implementation. High endorsement of open and reproducible practices coexists with lower self-reported adoption. Responses also emphasized the need for structural incentives and institutional support, reflecting perceived limitations in time, resources, expertise, and professional recognition.

## Introduction

Scientific reproducibility is crucial to scientific integrity and credibility, as it helps to verify how research data are generated and identify data manipulation or methodological flaws ( National Academies of Sciences, Engineering, and Medicine, 2019). Utilizers of scientific results (e.g., broader scientific communities, policymakers, clinicians, and the wider public) rely on scientific findings. If the results are not reproducible, it undermines trust and might lead to poor decisions in several areas, such as health, economics, and environmental regulation. Several studies have focused on unsuccessful replication attempts ( Begley & Ellis, 2012; Ioannidis, 2005; Munafò et al., 2017; Open Science Collaboration, 2015; The Brazilian Reproducibility Initiative et al., 2025) that increasingly brought the crisis narrative to the fore. Reproducibility is an important precondition and cornerstone of research quality ( National Academies of Sciences, Engineering, and Medicine, 2019), and has been widely discussed across various disciplines in the past decade, including psychology, medicine, economics, and biology ( Begley & Ellis, 2012; Ioannidis, 2005; Open Science Collaboration, 2015; Nosek et al., 2022).

There is a range of definitions for reproduction and related concepts such as replication and repetition, which diverge across fields ( Fidler & Wilcox, 2026). Any research study should, in principle, be reproducible by following the reported methods. A large portion of academic studies involve data collection and analysis. Over the last decades, a plethora of computational tools have emerged, aiming to facilitate or deepen investigations, or both. These tools can be applied to datasets of any scale, from limited samples to massive or complex data. However, merely rerunning data analyses can be challenging due to the variety of scripts, their logical sequences, software versions, computational environments, or computational power required. Thus, this study investigates aspects of computational reproducibility.

Currently, computational reproducibility has become critically important, as much of today’s research work relies heavily on digitalized data and computational tools throughout the entire research life cycle from research design, through computer-aided data analysis, to automated reporting tools. Computational reproducibility can be defined as “the ability to recreate results using the original data and code (or at least a detailed description of the analyses)” ( Crüwell et al., 2023), and, computational scientist as “an academic whose research has both code and data components” ( Stodden, 2010). This concept holds that “when you use the same data as in the published article, you can reproduce the same results” ( Lakens, 2022), “the data and the computer code used to analyze the data be made available to others” ( Peng, 2011), thereby enabling the evaluation and reuse of research outputs, data, and code by other researchers. Research code or research software, defined as “Software that is used to generate, process or analyses results that you intend to appear in a publication” by ( Hettrick et al., 2014), is crucial for the reproducibility of research. Research code commonly refers to case-specifically developed computer code, from a few lines to a professional package, used in the process of scientific or academic research to analyze data, simulate models, or process information. Therefore, the sharing and evaluation of research codes are of key importance.

Before the launch of our survey in February 2024, several survey-based studies have been conducted on reproducibility ( Baker, 2016), data management and sharing ( Tenopir et al., 2011; Van Den Eynden et al., 2016), computational reproducibility ( AlNoamany & Borghi, 2018; Stodden, 2010), as well as on discipline-related specificities, for example, computational reproducibility in computational biology ( Barone et al., 2017) and geosciences ( Cerutti et al., 2021; Reinecke et al., 2022). In terms of code and data sharing, the most comprehensive cross-disciplinary survey on computational reproducibility ( AlNoamany & Borghi, 2018) was conducted six years prior to our survey, predating significant developments in open science infrastructure, journal policies, and research funding mandates. Most related studies focused on specific disciplines, leaving space for further interdisciplinary research. Moreover, this study systematically examined a wide spectrum of barriers and enabling factors, from individual practices to institutional and systemic constraints, that shape computational reproducibility across various research contexts. Perceived barriers, needs, institutional and individual self-reported practices regarding computational reproducibility are subjective constructs that cannot be obtained and assessed by inspecting published code and data sets, alone. Therefore a survey was considered as an appropriate method for this exploratory study.

In current scientific research, replete with new digital tools and technologies in all disciplines, there is an emerging need for data and code reuse. Therefore, this exploratory survey aimed to (i) provide an up-to-date overview that captures the current landscape of researchers’ needs, barriers, incentives, support mechanisms, and practices across various disciplines, (ii) examine the awareness of reproducibility principles and their self-reported implementation in daily research workflows, with the ultimate goal of improving scientific reproducibility.

Accordingly, a survey-based study was conducted to address the following key research questions:

1. What are the main perceptions about practices that support computational reproducibility?
2. How are code and data shared during the publication?
3. What obstacles impede computational reproducibility in the practices of researchers?
4. Which methods and tools are frequently employed to support computational reproducibility?
5. How often do researchers attempt to replicate the studies of their peers, and how do they succeed?
6. How do the responses to the above questions vary based on career stage, academic discipline, geographical location, and related factors?

## Material and methods

The present study followed the Checklist for Reporting Results of Internet E-Surveys (CHERRIES) guidelines ( Eysenbach, 2004) for reporting results of internet e-surveys and reflects on all of its necessary elements in the current Method section (S4). The study protocol was registered on the Open Science Framework (OSF) prior to data collection (S1). The original and complete survey material, to ensure reproducibility, such as LimeSurvey files (forms), anonymized survey responses, and descriptive statistical analysis results are publicly available in Gelsleichter et al. (2024, see ‘Underlying data’ section), ensuring data integrity this folder is supplemented as a Zip file. Files that enhance the comprehension are placed under ‘Extended data’ section, including, study protocol (S1), survey questions (S2), open questions responses (S3), reporting guidelines (S4), and output analyses in:

- Jupyter Notebook (S5, .ipynb file extension): Recommended for quick visualization directly within the OSF; high graphical resolution.
- HTML (S6, .html): Recommended for offline navigation; includes a table of contents sidebar and opens directly in any web browser.
- PDF (S7): Use this version if .ipynb or .html files cannot be viewed.

### Survey design

An anonymous open survey was used to examine researchers’ awareness, attitudes, perceived barriers, and self-reported practices related to Open Science (OS) and computational reproducibility. Survey questions were developed in consultation with members of our consortium OSIRIS (Open Science to Improve Reproducibility in Science, funded by the European Union under the grant agreement 101094725), resulting in six sections with 35 questions, along 12 pages: demographic questions (6 questions), open science practices, supporting computational reproducibility (8 questions), data publishing (6 questions), data reuse (1 question), tools and code publishing (12 questions), and code reuse (2 questions). The first technical section of the survey aimed to assess the awareness and extent of the use of practices supporting open science and computational reproducibility. All items on research practices (data publishing, code and tool publishing, data and code reuse, and documentation) are self-reported: they record what respondents state they do, rather than behavior independently verified against their published articles, data sets, or code repositories. Throughout this paper, these items are referred to as self-reported practices, while the remaining items concern awareness, attitudes, and perceived barriers.

The survey was conducted online using the LimeSurvey, a free and open-source software ( LimeSurvey, 2025). Instead of sending the survey directly to each participant, it was shared on social media channels (further details are provided in the next section). This survey was designed with the possibility of breaking down or filtering answers according to screening questions (i.e., filled out by researchers, carrying out quantitative research). Since we did not have control over participants’ invitation, a concern was raised about the software’s technical level of participants; for example, if the survey becomes too complex at some point, they could answer improperly (just to move on) or drop the survey. To avoid this, skipping mechanisms were set in some questions, based on previous ones; for example, in the question of *Choose the characteristics that describe the kind of research data you generate;* if the participant responds that ***I do not produce data in my research**,* the system skipped ahead to the next section. This mechanism was implemented in ten questions, the following: 1.4, 2.7, 3.1, 3.2, 3.3, 4.1, 5.4, 5.5, 5.8, and 6.1. The respondents were able to edit their responses as backward navigation was enabled. Furthermore, because the survey was comprehensive and long, multiple fillings were not considered as an issue, so a checking mechanism was not implemented. To avoid personal data collection, IP checks and the use of cookies were not performed.

To give the participant the necessary background of certain (more complex/technical) items, aiming to avoid any skipping, most of the items carried explanatory ‘tooltip’ style messages along the survey, similar procedure as ( Reinecke et al., 2022). During the survey the definition of reproducibility was provided as “Computational reproducibility can be defined as the capability to reproduce the same data analysis results using the same data and methods as in the published article” which aligns with ( Leek & Peng, 2015; Lakens, 2022; Crüwell et al., 2023), as presented in the study protocol (S1). Replication studies were provided as “research attempting to reproduce the methods and findings of prior research” ( Open Science Collaboration, 2015).

The questionnaire was developed iteratively within the OSIRIS consortium by a multidisciplinary group with expertise spanning agronomy, computer/data science, geomatics/GIS, and medical/health research, and including career stages from doctoral researchers to senior investigators. Prior to launch, we conducted internal pre-pilot testing with consortium members and PhD students focusing on clarity, length, skip logic, and technical usability; this process led to edits (rewording, item reductions, additions). While not a substitute for a respondent pilot with external feedback, this diverse design and pre-pilot process mitigated construct and usability issues. A similar approach was done by Stodden (2010). According to the study protocol, the survey was designed to be online for three months (approximately), from 2024-02-23 to 2024-05-31. However, as the number of respondents was still limited, it was decided to extend it until 2024-09-30, spanning six months (approximately).

### Survey population and recruitment

In line with the registered protocol, the survey targeted researchers across disciplines who collects and analyze quantitative (usually digital) data with computational methods and tools. This broad definition encompasses researchers from the natural, applied, and social sciences. The survey aimed to provide a general overview of awareness, attitudes, perceived barriers, and self-reported practices in terms of computational reproducibility.

To reach out to this wide range of researchers in terms of geographic and scientific coverage, too, the survey link was shared via the official social media channels of OSIRIS (LinkedIn, X) and was re-shared by OSIRIS partners via flyers at scientific conferences ( iEMSs, 2024 and local events of Hungarian University of Agriculture and Life Sciences) and through blog posts on scientific community websites ( International Environmental Modelling and Software Society, 2024; Springer Nature Research Communities, 2024). A similar approach was used by AlNoamany and Borghi (2018). The survey distribution relied on volunteer sampling within the research community, which eliminated the need to maintain a sensitive database of names and email addresses, which would be necessary for direct email-based recruitment.

As an exploratory study using volunteer sampling via social media channels (associated with an open science consortium, OSIRIS), the survey is subject to self-selection bias. Respondents who encountered the survey through open science networks are, by definition, more likely to be already aware of and favorably disposed toward reproducibility principles. Self-reported practices may additionally be affected by social desirability and recall bias, and should be interpreted as reported practices rather than as audited measures of actual behavior. The survey prioritized breadth of perspectives over representativeness.

### Ethics and consent

The survey-related work did not contain any research study on humans (individuals, samples or data). Since it is about research practices and workflows in context of computational reproducibility, and study was designed as an anonymous one, not involving human subjects in a sensitive or identifiable way, with voluntary participation (no personal data or identifiable responses were collected, even IP was not collected, or any cookie was set), consequently we did not initiate and obtain ethical approval for that.

The list of questions was designed in multiple rounds within the OSIRIS consortium which ensured that no harm or risk is posed to respondents.

Respondents, clicking on the survey link were navigated to an introduction page, where detailed information about the study was provided, including the link to the study protocol and the full list of questions, in advance, before starting the survey itself. In possession of this knowledge, participants had the chance either to access the survey by checking the “I agree to take part in the research” box, or to leave it without any consequence. Accordingly, respondents took part with electronically checked written consent.

In addition, the GDPR (Hungarian University of Agriculture and Life Sciences) office was consulted and informed about the nature and content of the study. They verbally informed us that GDPR is not a relevant issue in the case of this particular survey. The detailed consent page can be found in the OSF survey material ( Gelsleichter et al., 2024, see ‘Underlying data’ section).

### Data analysis

The survey comprised closed questions, supplemented by six open-ended questions at the end of the technical sections. These open-ended questions provided free text space for respondents to express their views about the given questions.

Closed questions were analyzed by descriptive statistics, prepared using Quarto ( Allaire et al., 2022) version 1.6.32 within RStudio ( Posit team, 2025) version 2025.05.0+496. The R ( R Core Team, 2024) used in the analysis can be found in ( Gelsleichter et al., 2024, see ‘Underlying data’ section). Quarto made it possible to prepare the data analysis along with data visualization for the reporting materials, ensuring the reproducibility of the work.

Both quantitative and qualitative data were summarized using numbers and percentages. In the case of questions that asked respondents to select and rank options from predefined lists, a simple weighted scoring method was applied to evaluate ranking. Depending on the number of ranked items, rank 1 = 3 points, rank 2 = 2 points, and rank 3 = 1 point, as well as rank 1 = 5 points, rank 2 = 4 points, rank 3 = 3 points, rank 4 = 2 points, and rank 5 = 1 point, scoring was applied to convert rank percentages into a single composite score for each item (see ‘Extended data’ S8 for detailed calculations).

Open-ended questions were partly categorized for a more conscious evaluation and interpretation of opinions. Categorization was made by MV and discussed, and consensus was reached between MV and YAG. Complete replies to open-ended questions can be seen in (S3) under ‘Extended data’ section.

The questions were also analyzed using demographic group breaks. This analysis can be found in S5, S6, S7, under ‘Extended data’ section. Countries were categorized into two groups, developed and developing, based on the UNDP classification (2025).

## Results

### Demographics of respondents

The survey was initiated by 254 respondents; 194 of them (76.4%) completed the demographic stage and were distributed across the six inhabited continents: *Europe 157/194 (80%), followed by Asia 18/194 (9.3%), North America 8/194 (4.1%), South America 7/194 (3.6%), Africa 2/194 (1%), and Oceania 1/194 (0.5%)*. In total, 133 respondents completed the survey. The majority of the drops were immediately after the demographics; therefore, the study only considered 133 complete responses. The final sample maintained similar geographic distribution: *Europe 109/133 (82%), followed by Asia 8/133 (6%), North America 6/133 (4.5%), South America 5/133 (3.8%), Africa 2/133 (1.5%), and Oceania 1/133 (0.75%)* (S1: question 3). As the consequence of skipping mechanisms described in the survey design, the number of respondents varied across questions. Regarding the scientific field of respondents ( Table 1), it is distributed across *Natural sciences* 32/133 (24%), *Medical* 28/133 (21%), *Agricultural* 27/133 (20.3%), *Engineering & Technology* 26/133 (19.5%), and *Social sciences & Humanities* 20/133 (15%). In line with the target population defined in the protocol, respondents were mostly *Researchers and Academics* (120 of 133 respondents, 90.2%). For the career stage ( Table 1), most (50 out of 133 respondents, 38%) were *Established researchers* based on the EURAXESS (2023) classification, followed by *First stage researcher II* (defined as: carry out research under supervision, graduate students; 30 out of 133, 22.6%). Considering the type of institutions, 73/133 (55%) responses were from universities or higher education institutes, 40/133 (30%) from research institutes or research centers, 7/133 (5.3%) from non-profit organizations, 6/133 (4.5%) from government agencies or their departments, 2/133 (1.5%) from commercial entities, 1/133 (0.75%) from government operated commercial entities, and 4/133 (3%) from other types of institutions (university clinic, university hospital, scientific journal, or did not want to disclose). The target population of the survey was not only computer scientists, but also all disciplines utilizing digital data collection and analysis tools. The balanced disciplinary distribution broadens the range of perspectives represented; it does not, however, make the sample representative of any discipline’s wider population, given the volunteer, self-selected recruitment (see Study limitations).

**Table 1.** Demographics, composed of *type of institution, field of research, interest, career stage.*

Category	Subcategory	Count
*Type of Institution*	*University or higher education institute*	73
	*Research institute (or research center)*	40
	*Non-profit organization*	7
	*Government agency or department*	6
	*Other ^1^*	4
	*Commercial entity*	2
	*Commercial where the government is a major stakeholder*	1
*Field of Research*	*Natural sciences*	32
	*Medical sciences*	28
	*Agricultural sciences*	27
	*Engineering and technology*	26
	*Social sciences and Humanities*	20
*Interest*	*Researchers and Academics*	120
	*Other ^2^*	6
	*Journal and Publication Professionals*	3
	*General Public*	2
	*Policy Maker and Governance*	2
*Career Stage*	*Established Researcher*	50
	*First Stage Researcher II*	30
	*Recognized Researcher*	18
	*Leading Researcher*	17
	*First Stage Researcher I*	9
	*Other ^3^*	9

Meaning of Others in the Type of Institution ^1^: *University Clinic, University hospital, Scientific journal,* and *I do not want to disclose it.* Each had one reply.

Meaning of Other in Interest ^2^: *Data sharing support, Data steward, IT support, Research reform advocate, Research Support services,* and *Statistical analysis.* Each had one reply.

Meaning of Other at Career Stage ^3^: *Retired researcher, now coordinating editor; Software developer (worked for researchers for 10 years), Semi-retired researcher, Data steward, Support staff, Research software engineer.* Each had one reply and *I do not want to disclose it* had three replies.

To illustrate the nature and focus of the respondent population, in the checkbox Question 3.3, which asks what *kind of research data you generate,* among several listed options, *Quantitative data* (numeric files, survey responses, geospatial data), *Omics data* (information generated by studies ending with -omics: genomics, proteomics, phenomics, etc.), *Imaging data* were selected mostly (91%, 21%, and 21% among 120 respondents, respectively).

### Open Science practices supporting computational reproducibility

**Figure 1.**
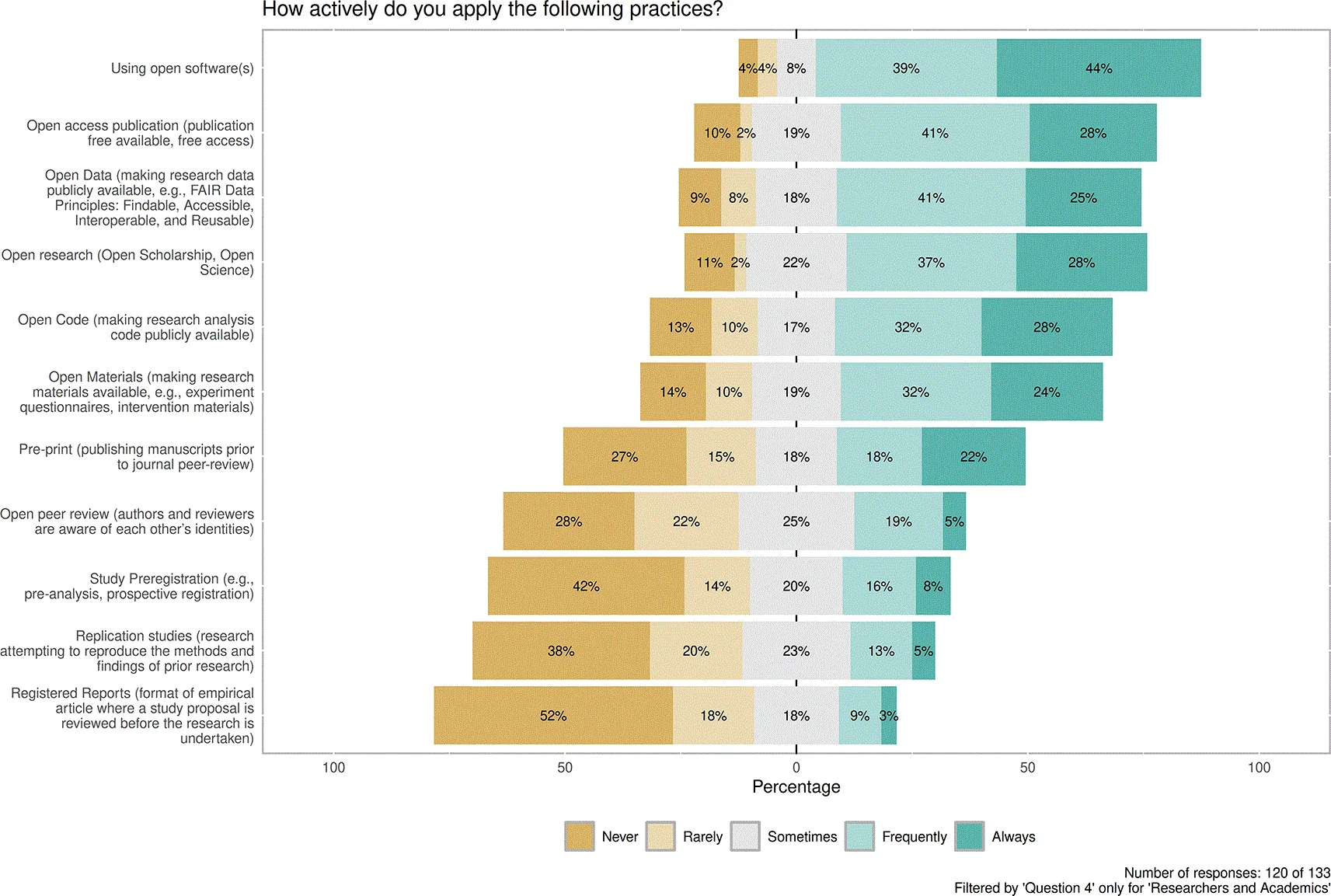
Application of open science practices. Contains the distribution between replies Never, Rarely, Sometimes, Frequently and Always in context of predefined practices. Number of responses: 120 of 133. Filtered by 'Question4' only for 'Researchers and Academics'.

Regarding the awareness and extent of use of practices supporting open science and computational reproducibility, a question focused on the *prevalence of various tools, methods and techniques, by listing 11 OS practices* to cover the available solutions as much as possible ( Figure 1). The *Use of open-source software* was widely self-reported **,** with 100 of 120 respondents (83%) indicating that they used it *Frequently* or *Always*. Followed by *Open access publication* with 83/120, 69% of *Frequently* and *Always* options suggests that it is also a widely known and used practice (meaning that “ *there are no financial, legal or technical barriers to accessing it*”), ( openaccess.nl, 2025).

*Open data*, *Open research (including open scholarship)*, *Open code and open materials* can be considered as less frequently applied practices among respondents with a range of 68–79 from 120, 56-66% of *Frequently* or *Always* replies.

On the less common side, *Registered reports*, *Replication of studies*, or *Study pre-registration* belong to less known and applied practices, where 62/120, 46/120, and 50/120 respondents (52, 38%, and 42%, respectively) reported *Never using* these practices. *Open peer review* was among the least common practices with 60/120 (50% of responses) when combining *Never* (34/120, 28%) and *Rarely* (26/120, 22%).

According to replies on the question about *Sharing of Data*, *Code* and *Research documentation* in case of work with public funding should be made accessible, according to respondents (96/133, 72%; 84/133, 63%; and 97/133, 73%, respectively). Although what is more notable here is the relatively high percentage of ‘neutral’ responses (37/133, 28%; 43/133, 32% and 31/133, 23%) which implies that there is still a great need to raise awareness and to develop incentive schemes.

**Figure 2.**
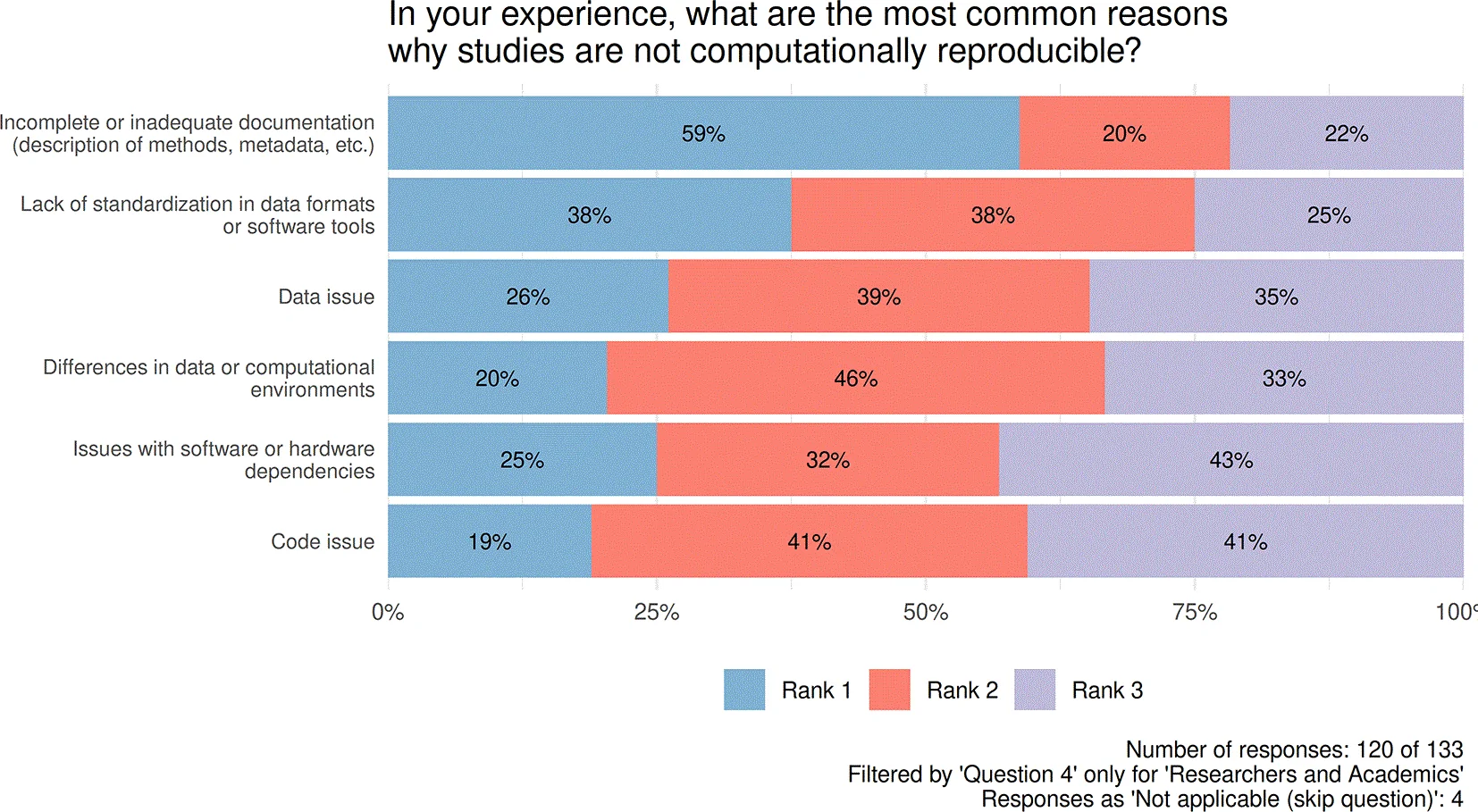
Ranked obstacles to computational reproducibility. Figure introduces the experiences about the most common reasons why studies are not reproducible. Respondents were asked to select and rank three items (Rank1, Rank2, Rank3) deemed the most important in their view. Number of responses: 120 of 133. Filtered by 'Question4' only for 'Researchers and Academics'.

In the question of revealing barriers to reproducibility ( Figure 2), respondents were asked to select and rank three items deemed the most important in their view. Based on the “Rank 1 = 3 points, Rank 2 = 2 points, Rank 3 = 1 point” conversion, *Incomplete or inadequate documentation* received a score of 287 (see ‘Extended data’ S8 for detailed calculations) at first place, followed by *Lack of standardization in data formats or software tools* with 258 points (S8), and *Data issue* with 229 points (S8).

In the degree of efforts made toward reproducing others’ work, 33/120 (27.5%) respondents reported *Never having attempted to reproduce a study*, while only 9/120 (7.5%) respondents reported *High degree of reproducibility*, compared to the 24/120 (20%) respondents with the opinion of *Low degree of reproducibility* or *Impossible to reproduce.* The number of replies with a neutral *Medium degree of reproducibility* was 53/120 (43.3%). Another question illustrates that according to experience, open data, open code or metadata, gathering *Never*, *Rarely*, and *Sometimes* appear in the publications studied by respondents with 70, 71%, and 86%, respectively.

**Figure 3.**
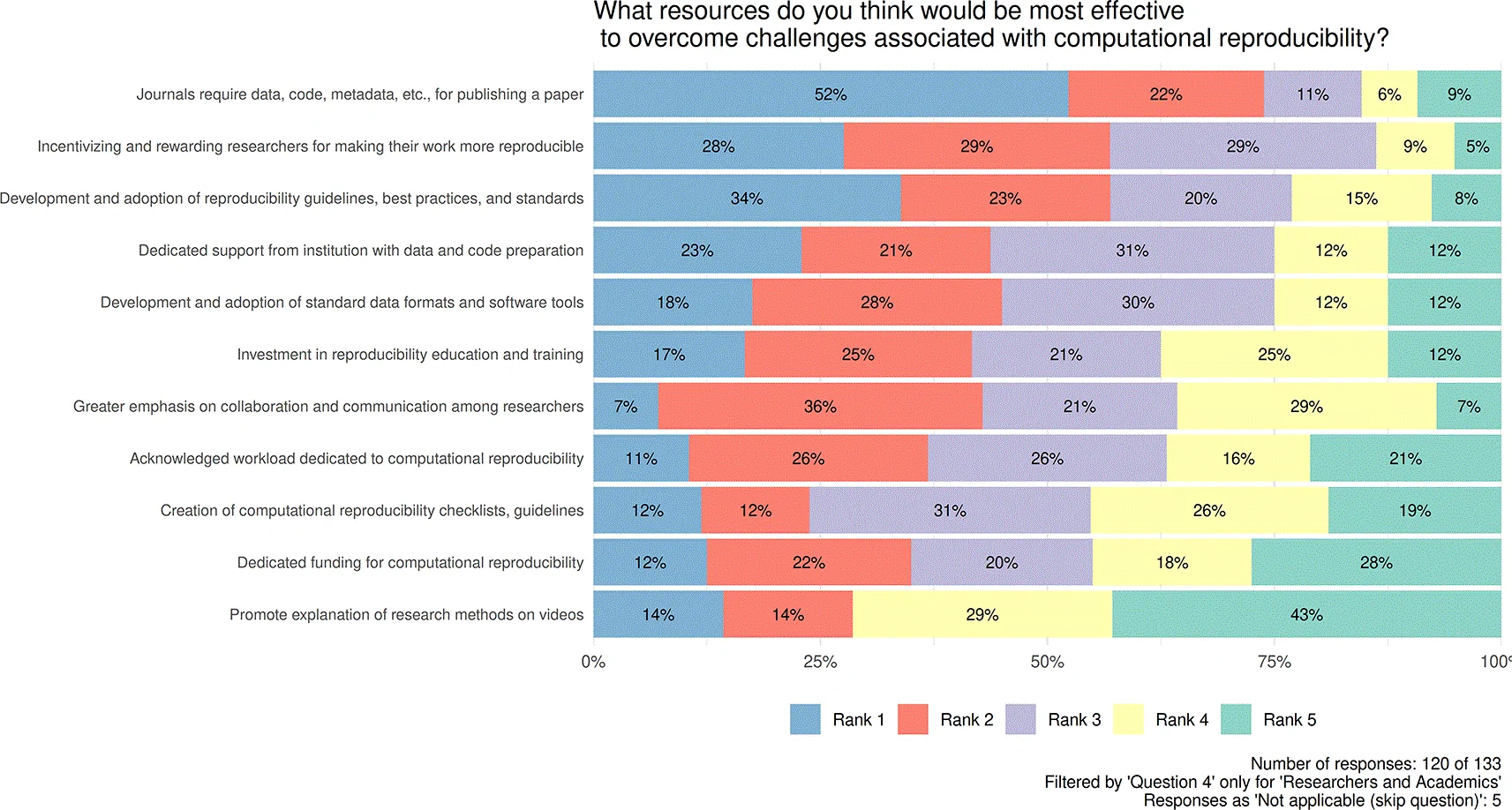
Strategies deemed important to overcome the challenges in computational reproducibility. Contains information about selection and ranking of up to five items (Rank1, Rank2, Rank3, Rank4, Rank5) that respondents deemed the most important from amongst the predefined list of 11. Number of responses: 120 of 133. Filtered by 'Question4' only for 'Researchers and Academics'.

Transparent and computationally reproducible research requires effort from researchers, accompanied by appropriate resources. A question to capture opinions about these resources was framed ( Figure 3), asking for the selection and ranking of up to five items that they deemed the most important from amongst the predefined list of 11 (note, *Not applicable* replies were removed from the analysis). Based on the responses of 120 academics/researchers and on the applied five-point scoring system, the most important driver is whether *Journals ask for the necessary data/code/metadata* (with scores of 482, S8). This is followed by *Incentivizing and rewarding researchers for making their work more reproducible* (with a score of 439, S8), followed by *Development and adoption of reproducibility guidelines, best practices, and standards* (with a score of 432, S8). In the shared 4th place, *Development and adoption of standard data formats and software tools*, as well as *Dedicated support from institution with data and code preparation*, both with a score of 394 (S8). The 5th item of the list is *Investment in reproducibility education and training* with a score of 372 (S8).

**Figure 4.**
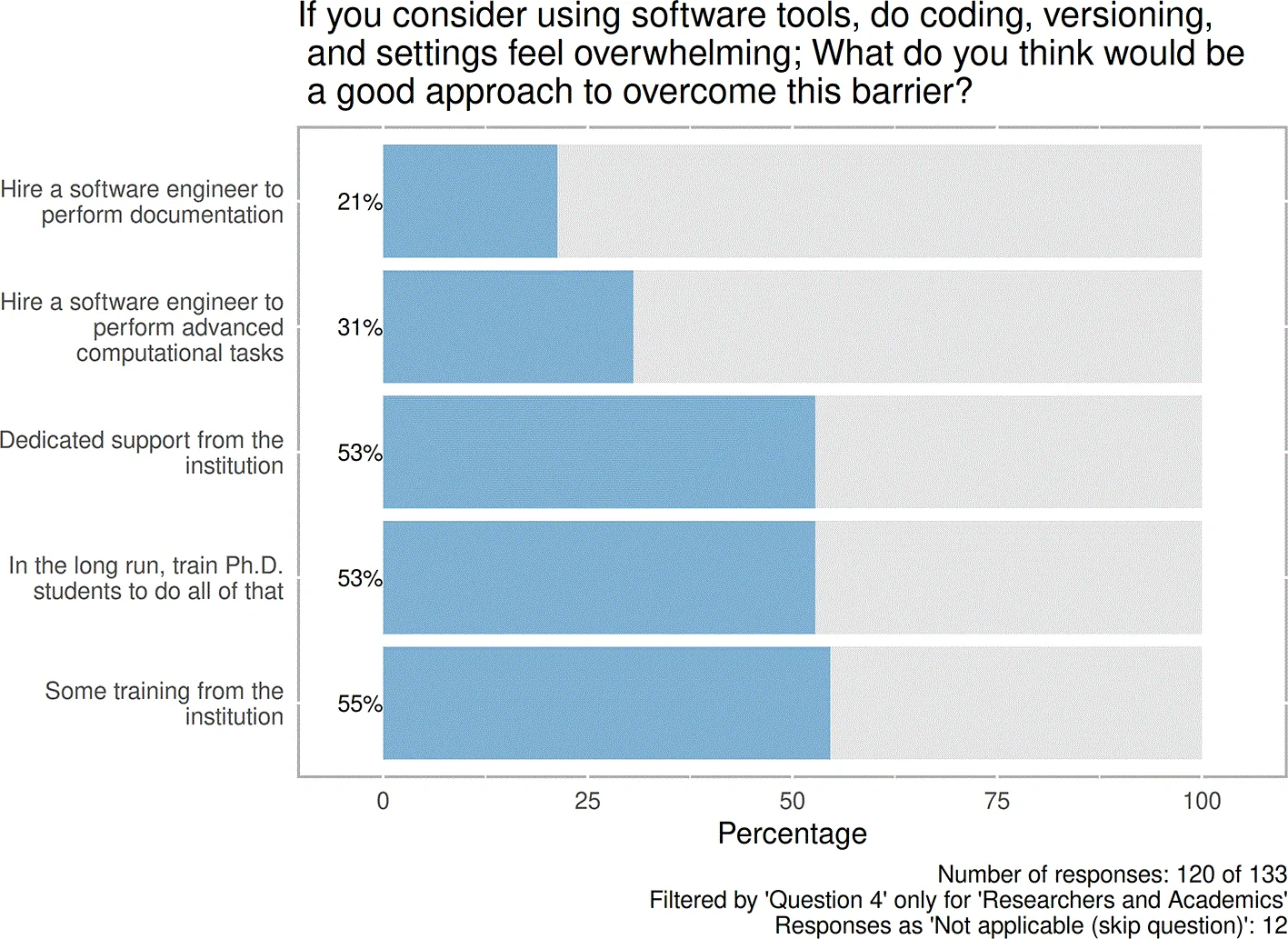
Actions to support computational work. Contains information about predefined and selected options that are considered important to overcome the barriers of computational reproducibility. Number of responses: 120 of 133. Filtered by 'Question4' only for 'Researchers and Academics'.

In addition, respondents were asked to check for the listed options that are considered important to overcome the barriers of computational reproducibility ( Figure 4). Among the five options, respondents highlighted the role of training in the first place, followed by the need for dedicated support, as well as training of PhD students in the long run (66/120, 55%; 64/120, 53%; and 64/120, 53%, respectively). In addition, the question offered an open-ended option, in which five opinions were shared. Four mentioned time, funding, and more conscious workflow management during the research process. One respondent expressed strong aspirations, namely “ *better criteria for job recruitment. If you cannot do this unsupported, you’re not supposed to be a researcher*”.

At the end of Section 2, an open field was provided to allow respondents to share their insights about good practices that could enhance computational reproducibility. Twenty-one respondents provided detailed opinions categorized into five main topics (with some insights):

1. Incentives from journals/funding agencies/institutions (8 replies)
*“Journals should not only require, but also review the code and data for each submission.”*
2. Actual technological solutions (4 replies)
*“Using declarative deployment systems like Nix/Guix to limit issues related to dependencies and ease deployment.”*
3. Importance of behavior change, focusing on early career researchers (3 replies)
*“We also need to train people how to write good code and document things.”*
*“I also believe journals need more methodology review experts to evaluate thoroughness of reporting.”*
4. Trainings, guidelines, hackathons for improving skills (2 replies)
*“Repro hackhaton.”*
5. Suggestions for dedicated positions (both at institutions and in journals) (2 replies)
*“Appoint Open-Science-Friendly engineers/researchers as referent within all research units to communicate with fellow researchers and convey good practices, with nationwide exchanges between referents within a national (possibly international) network.”*
*“Emphasize that it is a revolution, but it does not have to be all done right away => step-by-step process, project after project, improvements after improvements, mistakes are ok (even in codes and data). And not everybody can change how they do research at the same pace: list all that can be done and ask people what small changes they can do today? And what could they plan to do in the future?”*

Detailed responses can be found in the respective OSF repository (open questions responses (S3), under ‘Extended data’ section).

### Sharing research data

Considering the equilibrated range of disciplines that participated in the survey, qualitative data (interviews, focus groups, field notes, images, audio, video, etc.) were selected by 22% of respondents. *If I do not produce data in my research* or *Not applicable* was selected, the following answers were skipped and respondents were taken to the next section: In the survey, 33/120 (27.5%) respondents were skipped, and 87/120 (72.5%) went through.

Respondents were asked to estimate the number of shared datasets over the past five years. As a result, more than half of the respondents (70/116, 60.3%) reported *Practice data sharing in their own work* (the responses are distributed as follows: 64 replies with 1-10 datasets, 3 replies with 14 datasets, 1 reply with 20 datasets and two replies with more than 30 datasets).

Responses show the popularity of *Data repositories* (Zenodo, Dryad, Mendeley data, Figshare) with 56/87 (64%), followed by *Supplementary materials* 45/87 (51%), and *Data papers* (Data in Brief, Scientific Data, etc.) with 32/87 (37%) among researchers who reported practice data sharing. As this question was a checkbox selection, participants were able to choose more than one option; therefore, each item had a hundred percent possibility. Other free box options also highlighted the option of GitHub, the OSF repository, and the project websites of funding bodies.

**Figure 5.**
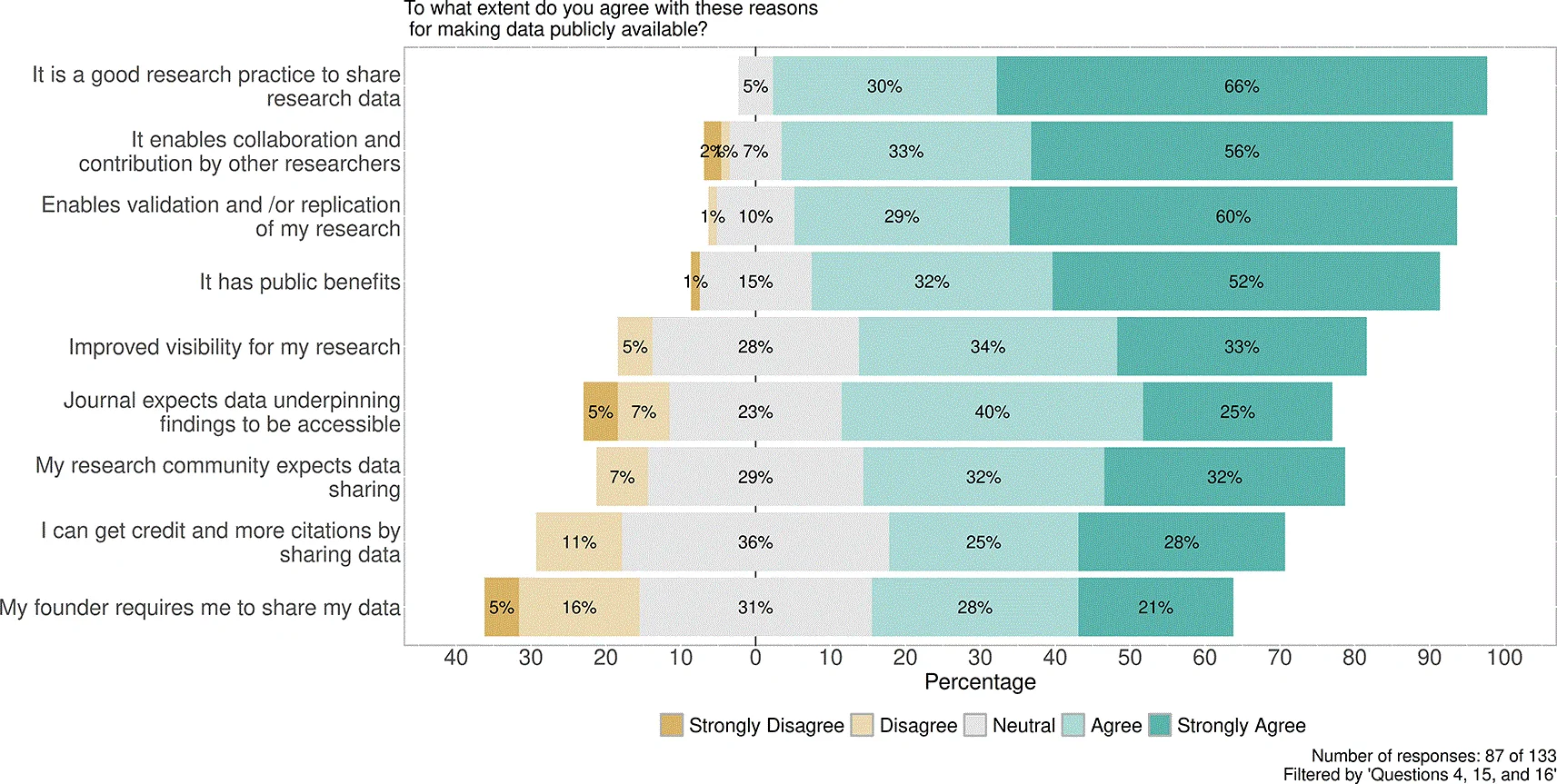
Reasons to make data publicly available. Contains the distribution between replies Strongly disagree, Disagree, Neutral, Agree and Strongly agree in context of the question why making data publicly available is important. Number of responses: 87 of 133. Filtered by 'Questions 4, 15 and 16'.

In response to the question ( Figure 5) *why making data publicly available is important*, different motivations were grouped by the level of agreement. *Because it is a good research practice* 84/87, (96%) respondents *Agree* (30%) and *Strongly Agree* (66%). At the same time, 77/87 (89%) respondents considered it important for *Enabling collaboration and contribution by other researchers*, *Agree* (33%) and *Strongly Agree* (56%), and close ratios for *Enabling validation and replication* (89%) when combining *Agree* (29%) and *Strongly Agree* (60%), and *Public benefits* (84%) when combining *Agree* (32%) and *Strongly Agree* (52%). Respondents agree or strongly agree with the options of *My funder requires* and *I can get credit and more citations* with 49/87 (56%) and 57/87 (65%), respectively.

**Figure 6.**
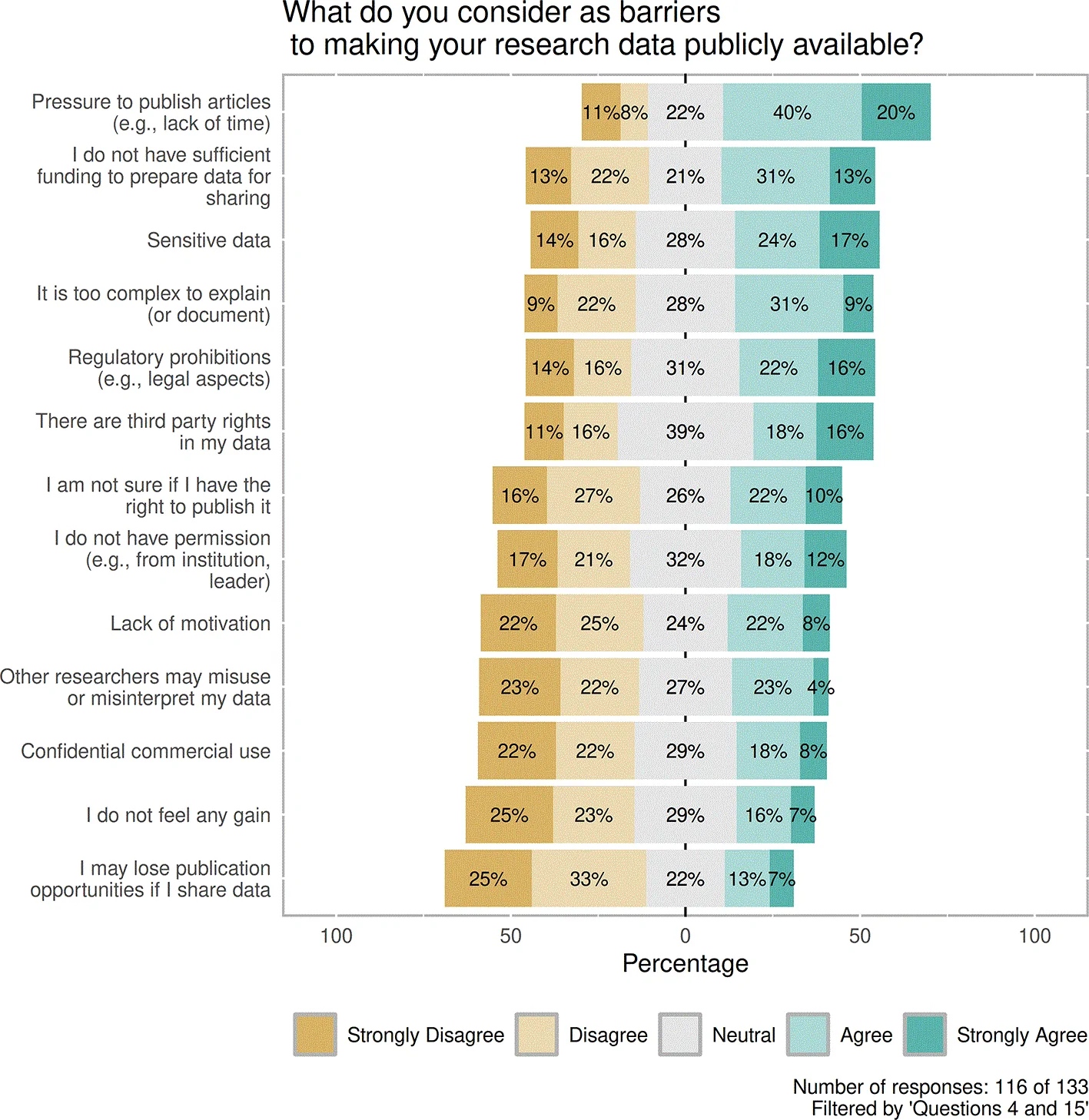
Barriers to making data publicly available. Contains the distribution between replies Strongly disagree, Disagree, Neutral, Agree and Strongly agree in context of predefined barriers. Number of responses: 116 of 133. Filtered by 'Questions 4 and 15'.

Considering the barriers ( Figure 6), respondents were asked to judge 13 predefined reasons that hindered data sharing. Here, the most common reason was the lack of time (i.e., the pressure to publish articles) (70/116, 60%), followed by the lack of sufficient funding that supports data sharing (51/116, 44%), and the sensitive characteristics of data were mentioned in third place (48/116, 41%) with *Agree* or *Strongly Agree.* Data complexity, uncertainty about rights to share, and lack of permissions can be considered moderate barriers, with a relatively high ratio of neutral responses, suggesting some level of perplexity. Factors of *Losing publication opportunities*, *Feeling of additional gain*, *Confidential commercial use* or *Lack of motivation* with a relatively high number of *Strongly Disagree* and *Disagree* options (67/116, 58%; 57/116, 48%; 51/116, 44%; and 52/116, 45%) show that these cannot be considered as major barriers.

Under the point of view of career stage ( *Always+ Frequently*), *First stage researcher I* pointed several reasons to avoid data sharing, such as not having permission, funding, loosing opportunities, and too complex to explain. However their level of motivation is not far from other career stages (S5, S7: question 19).

At the end of Section 3, 11 comments/opinions arrived, categorized into the following three topics, accompanied by meaningful replies (some examples are highlighted):

1. Regulations and requirements about data sharing (4 replies)
*“Simply it is not a requirement by education institutions. Otherwise lots of data would be available. And data sharing habit would start right away at university”.*
2. Technical issues (3 replies)
*“Make sure institutions/governments do not invent their own “data license” that is then hard to interpret.”*
*“Perfectionism (not wanting to publish data that is not processed/cleaned perfectly).”*
3. Behavior change (1 reply)
*“All publically funded research should require the data published.”*

Detailed responses can be found in the respective OSF repository (open questions responses (S3), under ‘Extended data’ section).

### Data reuse

Regarding the reuse of existing data in Section 4, the most commonly mentioned purposes of utilization are for research validation, providing background or context to the given actual research, to reuse them in the development of their own methodology, and for teaching material. The relatively low response rate (30%) for replication and meta-analysis might also originate from definitional difficulties despite the explanatory pop-up messages of the survey. In the *Other* open box option, respondents also mentioned crowd-science projects, systematic reviews, or derivates data (e.g., maps from point data were actually referred). Only 6% of the 120 respondents stated that they had never used existing data. This supports the case for promoting data sharing, responses, and individual quotes to outline the actual state of data sharing and reuse and also implies the need for harmonized expectations that would most likely be highly supportive in terms of making data openly available (or at least accessible in line with the FAIR principles; Wilkinson, et al. (2016) as a norm in the research process.

### Insights about tools and code publishing

The aim of the respective question was to discover the extent of the use of different types of digitalized tools for data management and analysis. Considering the replies, R programming, and conventional spreadsheets (62/120, 52% of respondents stated that they use them *Always* or *Frequently* in both cases) are on the most popular side. In the middle range, Python programming language and various types of statistical software (SAS, SPSS, JASP, PSPP, GRETL, SOFA, KNIME, Scilab, etc.) were mentioned with 38/120, 32% and 36/120, 30%, respectively, considering *Always*+ *Frequently.* On the other hand, the least applied methods/tools are various, specific skill-requiring programming languages, programming platforms, and database management (68/120, 57%; 68/120, 57%; and 60/120, 50% reported never using them, respectively). Still, 28/120 respondents (23%) reported using analogical data collection. From the breakdown, considering Always+ Frequently, by development status respondents from developing countries reported much higher reliance on paper and spreadsheets, using around three times more paper and nearly twice the number of spreadsheets and statistical software (e.g., SPSS), while more developed countries use more programming languages (e.g., R and Python). By field of research, Agricultural sciences is the most analog of the fields (paper or notebook notes), followed by Engineering, Medical, Natural sciences and Social and Humanities, the latter is the most digital field (S5, S7: question 22).

Consistent with this pattern, the use of version control, and code repositories was reported more frequently among respondents from developed countries (23 and 25-percentage-point difference respectively), when compared to developing contexts (S5, S7: question 29).

In addition to the eight predefined groups of tools, the next open-ended question aimed to identify other options. Here, seven additional responses arrived, highlighting mostly individual tools, for example, AI-based tools for preliminary analysis, various workflow tools, and application programming interfaces (in general, not further defined).

In a next question, respondents were asked to share their insights on whether the research code should also be evaluated in the peer review process by checking the most appropriate reply from ten predefined options. Twenty-one respondents selected *I do not know* (21/120, 18%), suggesting uncertainty about the topic.

Furthermore, most of the open-ended responses suggested that research code should be checked in various ways. Respondents indicated that code checking could be performed by a quick visual inspection (18), by machines (17), by a human staff of the journal (15), by a third-party operated cloud (13), by a human researcher reviewer (13), or by a human from the journal staff, but only through a quick visual inspection (3). In comparison, 13 respondents believed that the code should not be checked.

To discover ways of utilizing the research code, respondents were asked to check multiple predefined options that apply to their activities. Accordingly, 109/120 (91%) reported using it for data analysis; 103/120, 86% for visualization; 88/120, 73% for data cleaning; 84/120, 70% for the automation of the research process; 84/120, 70% for the organization of data and research work; 67/120, 56% for the collection of data; and 52/120, 43% for communicating the research work. In case *I do not use any code* 7/120, 6% were selected, further questions were skipped, and the survey was completed, ready to finish, and submitted.

To check the extent of sharing research codes, the next question aimed to gain insight into the number of shared research codes in the past five years, where, similar to data sharing activities, respondents were asked to type a number. Of the 113 people responded, 45 reported never having shared codes, 55 reported sharing 1–10, and 13 reported sharing more than 10 codes.

Mostly cited reasons to *making research code publicly available* are the followings:

• 70/74, 95% *Good research practice,* where *Agree* (26%) and *Strong Agree* (69%).
• 63/74, 91% *Enables validation and/or replication,* where *Agree* (26%) and *Strong Agree* (65%).
• 65/74, 88% *Enables collaboration and contribution by other researchers,* where: *Agree* (23%) and *Strong Agree* (65%).
• 64/74, 87% *Public benefits,* where *Agree* (30%) and *Strong Agree* (57%).

In the respective open-ended questions about code publishing, some of the challenges were highlighted, along with a claim for uncertainty about the question. A respondent raises attention to the point that code is useful only if it is “nice and tidy,” however, to make it such, it requires programming (coding) training, experience, and considerable time for cleaning and checking, commonly scarce resources in academia, unfortunately.

Considering code-sharing and publication practices in Question 5.8, replies were filtered by the number of shared codes in Questions 4.1, 5.4, and 5.5. Accordingly, 60/74 (81%) replies indicated that most of the respondents who shared code *Use comment options to explain the code* parts and document all steps in the script. A similar ratio *Use README file for explanation* 59/74 (79%) or code publishing platforms (GitLab, Bitbucket, GitHub, etc.) to make the code publicly available 52/74 (70%). Version control was underrepresented by 41/74 (56%), despite its importance in code development. Specific code publishing platforms (like MethodsX, SoftwareX, etc.), as well as community or educational networks, are not typically used (5, 4%, and 3%, respectively) among respondents. Under *Other options* free text box, OSF, R Markdown, and Software Heritage Archive were also mentioned as applied solutions.

**Figure 7.**
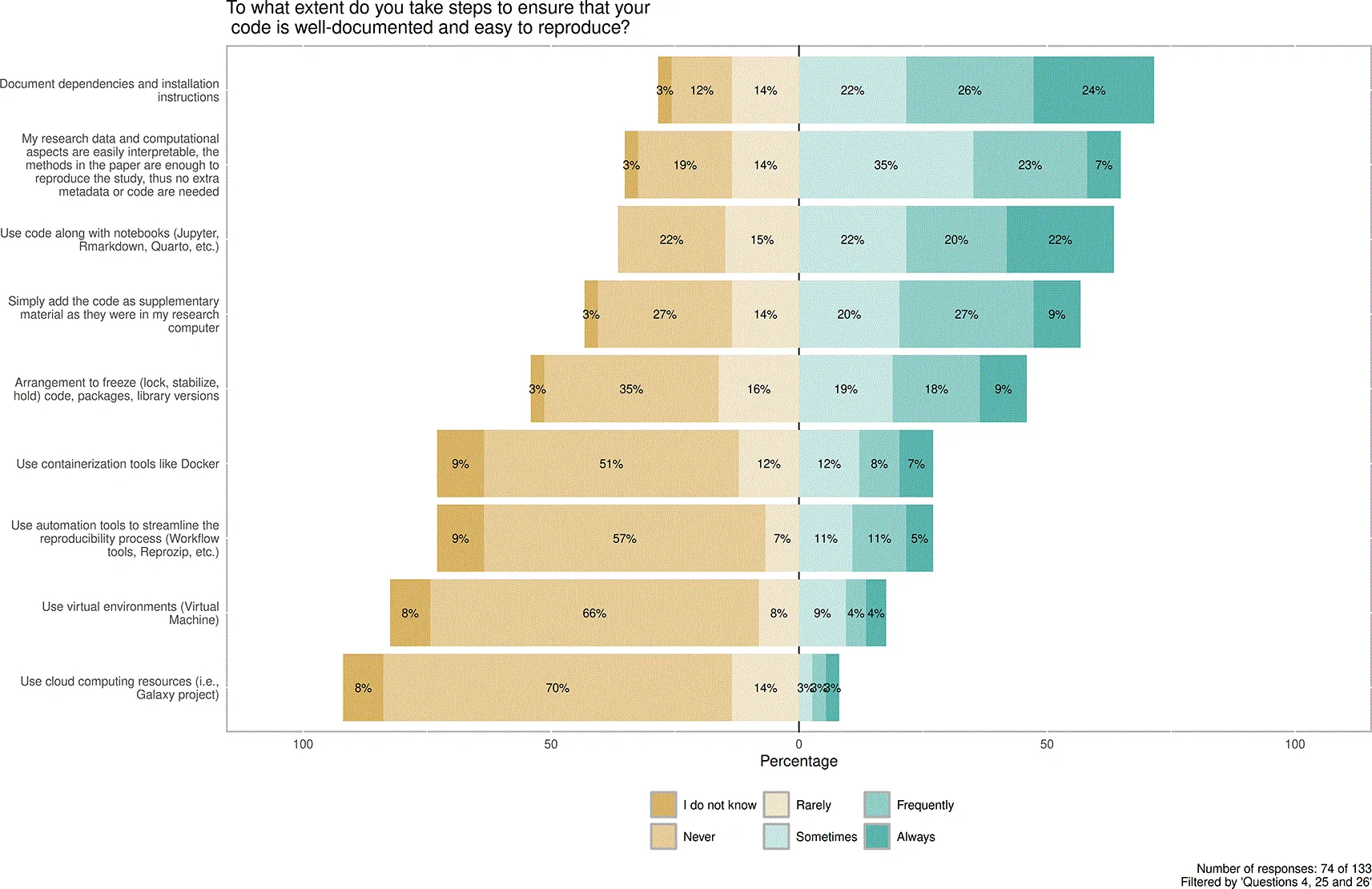
Actions made to enhance reproducibility. Contains the distribution between replies Do not know, Never, Rarely, Sometimes, Frequently and Always in context of predefined steps to making code well-documented and reproducible. Number of responses: 74 of 133. Filtered by 'Questions 4, 25 and 26'.

Continuing with the well-documented and easy-to-reproduce characteristics of shared codes ( Figure 7), respondents were asked to decide on a Likert scale (using *Always, Frequently, Sometimes, Rarely, Never*, plus *I do not know*) about efforts. On the most applied side, *Documentation of dependencies and installation instructions* (53/74, 72%) for *Always, Frequently* and *Sometimes* combined), and *Using code along with notebooks* (48/74, 65%) for *Always, Frequently* and *Sometimes* combined) are marked as applied practices. Among respondents who shared code, the majority reported never using cloud computing resources (52/74, 70%), virtual environments (49/74, 66%), automation tools (such as workflow tools or Reprozip ( Chirigati et al., 2016)) (42/74, 57%), or containerization tools (such as Docker ( Merkel, 2014)) (38/74, 51%).

For the respective open-ended questions about additional *aspects of code documentation*, seven replies were received. One part mentioned utilized tools (e.g., Guix, R script), and another part of replies highlighted the challenges of computational work:

• *“I usually only write R scripts and comment them, I feel I should do more with version control but I do not…”*
• *“I am aware of most of these tools and how it could/should be done (with containers etc.), but I never ventured so far, because my studies are not so general that I think anyone would touch it.”*
• *“Often projects have a combination of pipelines and notebooks this makes it very messy to share and takes time to organise but it is possible.”*

**Figure 8.**
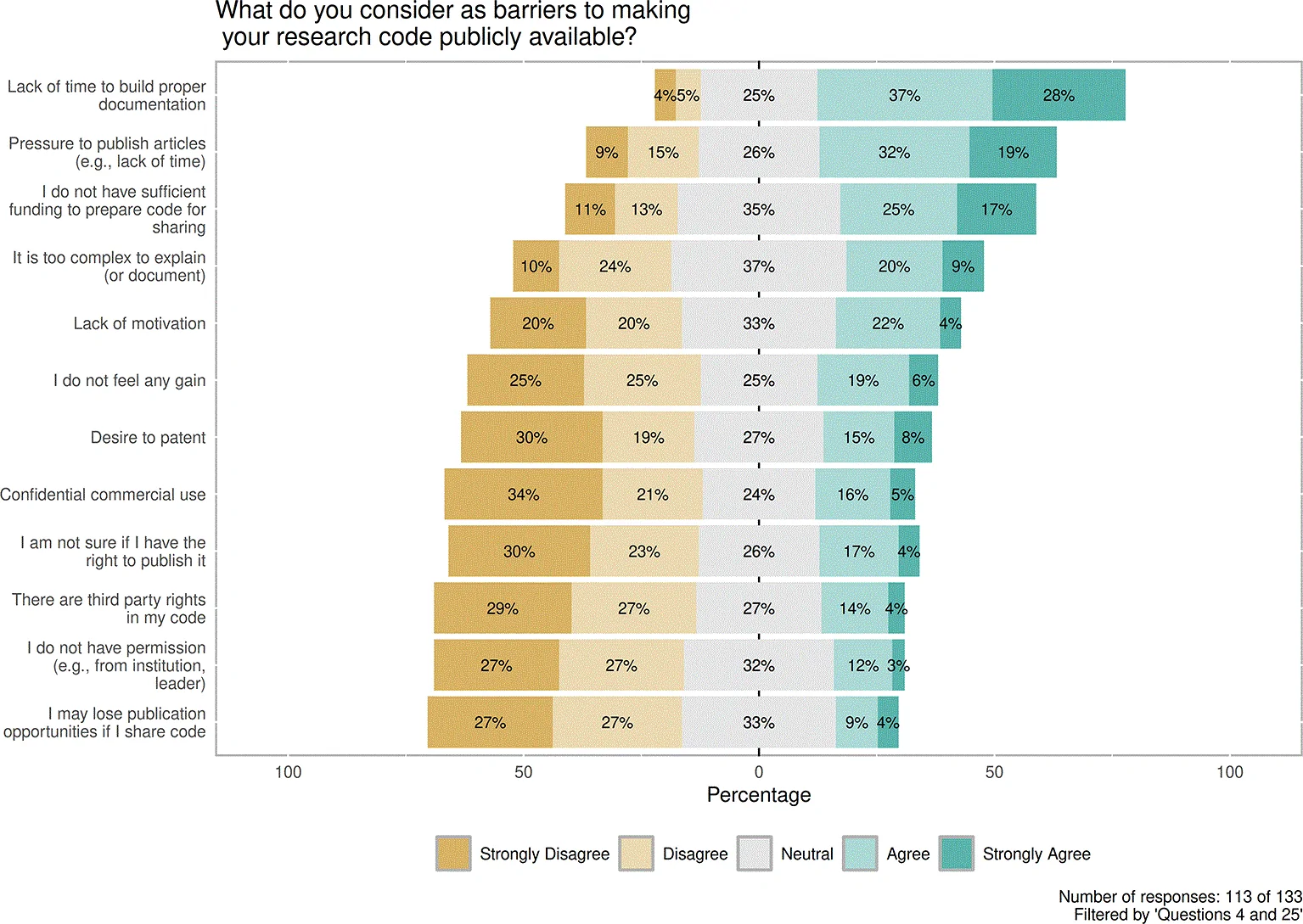
Barriers to making code publicly available. Contains the distribution between replies Strongly disagree, Disagree, Neutral, Agree and Strongly agree in context of predefined barriers to making code publicly available. Number of responses: 113 of 133. Filtered by 'Questions 4 and 25'.

Although respondents were aware of *Good research practices*, these replies were projected to the subsequent question ( Figure 8) about *barriers to making research code publicly available.* Fully in line with these highlighted free-text opinions, among predefined barriers: the *Lack of time to build proper documentation* 73/113, 65% ( *Agree* (37%) and *Strong Agree* (28%)); the *Pressure to publish* 58/113, 51% ( *Agree* (32%) and *Strong Agree* (19%)); and *insufficient funding to prepare code for sharing* 47/113, 42% ( *Agree* (25%) and *Strong Agree* (17%)) are the most commonly reported reasons. The following ones are not considered barriers, as respondents voted with *Disagree* and *Strongly disagree*: *I may lose publication opportunities if I share code* and *I do not have permission* both with 61/113, 54% ( *Disagree* (27%) and *Strongly disagree* (27%)).

The open-ended question of the section provided space to add aspects of the limitations of code-sharing and publishing. In this question, six open-text replies arrived. The following three highlighted opinions raise points about the previously mentioned barriers:

• *“There are activities that are more highly valued that i do instead (publishing).”*
• *“I have to actively fight my PhD advisor to package my code with guix and really document the deps down to the kernel, because he reckons a requirements.txt is enough and I’m wasting the project’s time.”*
• *“To me, publishing the code is a way to document the scientific process. Unfortunately, some people expect the code to be reusable without any effort (to analyze another dataset for instance), which is not the purpose of publishing the code (publishing a package/software is a totally different process). This misunderstanding often leads to uninteresting email debugging discussions, which could discourage a “full publication” strategy of the research code.”*

### Insights about code reuse

Regarding the reuse of the research code, respondents were asked to check all the options that apply to their practices. Accordingly, a high proportion of respondents reported using existing code to improve their own research code or to learn new coding strategies (86/113, 76% and 75/113, 66%, respectively). Research validation, replication, or utilization as a teaching material was in the range of 46-51 respondents out of 113, 41-45%, while 15/113 (13%) did not reported to use existing research codes. Considering the credibility of reused research codes, researchers deem open accessibility and well-documented codes crucial.

Regarding factors considered important when using existing code, the most frequently reported factors were well-established documentation (84%) and open-access code (83%), followed by obtaining code from a reputable source (56%). Clearly defined rights to use the code, references to the code in research papers, immediate access, and the possibility of citing the code were selected less frequently, by 43%, 50%, 54%, and 45% of respondents, respectively.

## Discussion

Discussion is organized following the structure of research questions.

### Perceptions about practices that support computational reproducibility

Key message: Researchers associate reproducible practices with transparency, collaboration, and public benefit. While awareness of the importance of reproducibility was widespread among respondents, their stated adoption of some reproducibility practices was less consistent, and many were cautious about sharing before publication.

When asked why making data publicly available is important, 84/87 (96%) of respondents agreed or strongly agreed that it constitutes good research practice.

Social desirability bias cannot be ruled out in responses to such normatively framed questions ( Fisher, 1993). Nevertheless, 77/87 respondents (89%) also agreed or strongly agreed that public data enable collaboration and contributions by other researchers. Similar levels of agreement were found for enabling validation and replication (89%) and providing public benefits (84%).

Responses concerning the public availability of research code showed a similar pattern. Together, these findings indicate strong stated support for sharing data and code, but do not demonstrate consistent engagement in these practices. The results highlight a gap between awareness and the self-reported adoption of reproducible practices. As pointed out by ( Stodden, 2010), researchers may be reluctant to share work before publication to protect their ideas while the work is still in development. In the same perspective ( Tenopir et al., 2011), found that only 30.5% of the scientists agreed to share data before publication.

On the other hand, the least indicated reasons for researchers sharing their code are the funder requirements, as well as obtaining more credit and citations. This ordering suggests that respondents placed greater emphasis on the perceived scientific benefits of code sharing than on external requirements or rewards. However, considering resources ( Figure 3), respondents ranked journal requirements first as they would create equal requirements and established expectations, thereby promoting reproducibility. This apparent difference may reflect the distinction between respondents’ stated reasons for sharing code and the external measures they believed could facilitate or normalize such practices.

According to the replies, when the study received public funding, more than 60% of participants agreed to share Code, Data and Documentation. A similar point was noted by ( Tenopir et al., 2011), publicly funded research must be public property. In contrast, it is interesting to point out that, in this research, nearly 20% of the leading researchers disagreed with sharing research documentation when looking at the demographic breakdown. This finding may reflect perceived time and workload demands, associated with preparing research documentation, although survey did not directly establish the reasons for this subgroup difference. Accordingly, behavioral change and education in the context of OS practices are still strongly needed.

### How code and data are shared during publication

Key message: Open-source software and open-access publishing were among the practices that respondents most frequently indicated using, while open peer review was less common and may depend partly on journal policies rather than on researchers’ individual decisions.

Open-source software is a key element in many tools and services, and it inherently supports reproducibility. As ( NI4OS-Europe, 2023) states, “the software-based services and infrastructure of OS are so important that it is safe to say that OS would not exist today without software, and, for a large part of that claim, without free and open-source software.”

The results show that the use of open software was adopted by 100/120, 83% (Frequently + Always) ( Figure 1) of respondents, demonstrating that the participants aligned OS practices with free software.

Scientific communication is moving toward a new stage defined by transparency and reproducibility ( Stodden, 2010). The Association of Scientific, Technical & Medical Publishers (STM) found that the proportion of gold and green open-access publications increased from 20% in 2013 to 43% in 2023 ( STM, 2025), across articles, reviews, and conference papers. In our survey, open access publication was in the second position among the most commonly used practices with 83/120, 69% (Frequently + Always in Question 2.1, Figure 1). However, Open peer review is a less common practice, mostly outside the researcher’s control. The results also highlight that other actors in the research ecosystem, such as journal publishers, play an essential role in disseminating good practices.

### Obstacles impeding computational reproducibility

Key message: Respondents ranked incomplete or inadequate documentation as the leading obstacle to computational reproducibility. Other frequently identified barriers included insufficient standardization, the time and effort required to prepare materials for sharing, publication pressure, limited incentives, and insufficient institutional support. These findings reflect the perceived priorities of this self-selected, open-science-engaged sample.

Incomplete or inadequate documentation is the top-ranked reason why the studies are not computationally reproducible. This finding is consistent with previous studies identifying missing documentation, files, and dependencies as major barriers to computational reproducibility. In a large-scale audit of more than 9,000 R scripts from over 2,000 replication datasets, 74% of the scripts initially failed to run in a clean environment; this proportion decreased to 56% after automated code cleaning ( Trisovic et al., 2022). A similar aspect, lack of documentation, was found by Reinecke et al. (2022). Together, these findings illustrate that making code available is necessary but may not be sufficient. Code and data must also be adequately organized and documented to support re-run and reuse.

Among the measures that could help overcome these barriers, respondents ranked journal requirements concerning data, code, and metadata first (score: 482; Figure 3), followed by incentives and rewards for reproducible work (score: 439; Figure 3). These rankings represent the priorities expressed by the surveyed group rather than evidence of the effectiveness of these interventions.

Aligning data and code management with the FAIR Guiding Principles (Findable, Accessible, Interoperable, Reusable) provides a concrete framework for improving documentation, metadata quality, and standardization, thereby lowering practical barriers to re execution and reuse ( Wilkinson et al., 2016).

Previous studies have identified a similar combination of technical and structural barriers, including inconsistent documentation and code-management practices, time constraints and insufficient incentives ( AlNoamany & Borghi, 2018, Liu & Salganik, 2019, Reinecke et al. 2022, Kedron et al. 2024). Shared executable workflows and standardized computational environments have been proposed as potential ways to reduce some of these barriers ( Cerutti et al., 2021). However, computational reproducibility cannot always be achieved through technical standardization alone, as discipline-specific limitations may also affect what can be reproduced ( Hocquet and Wieber, 2021).

Because these studies used different samples, questions, and analytical approaches, they do not support direct conclusions about changes over time. Nevertheless, the recurrence of similar concerns suggests that documentation, standardization, time, and incentives remain important perceived barriers across research contexts.

Among barriers to making research codes publicly available, respondents placed the first lack of time, followed by pressure to publish. Similarly, Stodden (2010) found that the largest barriers to sharing code are the time to clean up and document it for release, followed by issues in the code by other users. Tenopir et al. (2011) pointed out lack of time followed by lack of funding, while Gomes et al. (2022) found that the most common obstacles include the time needed to clean and document code, concerns about potential misuse or misinterpretation, and misaligned career incentives. Improper documentation is also confirmed by Reinecke et al. (2022).

The responses suggest that preparing data, code, and documentation for sharing is perceived as requiring additional time, effort, and support. The scale of this burden is likely to vary across projects. Routine analyses may be made reproducible through clear directory structures, basic documentation, non-proprietary tools where relevant, and deposition in trusted repositories, while sensitive data, complex computational pipelines, and proprietary dependencies may require substantially greater resources ( Stodden, 2010; Tenopir et al., 2011; Trisovic et al., 2022).

The survey cannot determine whether the perceived burden primarily reflects technical complexity, insufficient institutional support, or a lack of professional recognition. Nor can it establish the effectiveness of particular policy interventions. However, the responses indicate that technical guidance alone may be insufficient without appropriate incentives and institutional support. Future studies combining surveys with audits of published data and code could distinguish perceived barriers from those encountered during actual reproduction attempts.

### Replication practices and success rates

Key message: Almost one-third of the respondents reported that they had never tried to reproduce another study. Based on the identified reason, time and labor constraints limit researchers’ ability to invest in replication. Respondents also indicated that open data, open code, and metadata are rarely or only sometimes available in the publications they read (70%, 71%, and 86%, respectively). Respondents suggested that replication would be more successful if it was incentivized and better integrated into everyday research practices.

The survey also aimed to examine respondents’ attempts to reproduce the work of others. Importantly, 33/120 (27.5%) respondents reported never having attempted to reproduce a study.

In comparison with “59% of all participants never ran somebody else’s model to reproduce their results” from ( Reinecke et al., 2022), although their research focused on earth sciences researchers, instead of a broad target group like the present study. Consequently, these numbers suggest another intervention point to encourage reproducibility studies. However, considering time and labor constraints, it should be done and incentivized in a reasonable manner, linked strongly to actual research work. According to respondents’ experiences, Open data (84/120, 70%), Open code (85/120, 71%), or Metadata (103/120, 86%) were Never, Rarely, or only Sometimes available in the publications they are read.

## Study limitations

Given the method of distribution, we did not have information about either the reached population or the response rate, and non-response bias cannot be assessed. The demographics of the respondents who responded to the survey are described in detail in the Demographics section; this documents who answered, but does not establish representativeness.

The survey’s length (35 questions) and page navigation may have introduced fatigue effects, potentially reducing response quality for later items despite skip-logic and back-navigation.

As this exploratory survey was distributed openly through the social media channels, blog posts, and conference materials, including those of an open science consortium, recruitment was closely tied to the very characteristic under study, namely engagement with open science and computational reproducibility. The volunteer sampling via social media can induce self-selection bias among the participants, which can further inflate estimates of awareness and positive attitudes toward reproducibility. The observed prevalences are correspondingly high: frequent or constant use of open-source software was reported by 83% of respondents, and at least occasional use of registered reports by 48%, above what would be expected in an unselected sample of researchers. Accordingly, the findings may not reflect the broader research community, especially because demographics (mainly region) of respondents cannot be considered representative, as most replies arrived from Europe (109/133, 82%); the generalizability of the findings is unknown even with respect to the open science-aware population from which the sample is drawn. The same applies to the ranked barriers and enabling factors ( Figures 2, 3, 6, and 8), which reflect the priorities of this group; researchers not engaged with open science may identify different obstacles altogether.

Despite these limitations, self-selected open science-aware samples are themselves an important target group for understanding which barriers persist even among motivated researchers; findings in this stratum are particularly informative for policy design aimed at the remaining structural obstacles. Selection on open science engagement biases estimates upward, which makes low observed adoption informative in one direction: practices that remain uncommon here, such as registered reports, replication, and open peer review, are unlikely to be more common in the wider research population. The barriers reported recur across the disciplinary groups represented, indicating that they are not confined to a single field within this stratum, and they align with those identified in similar previous studies (e.g., Liu & Salganik, 2019; Reinecke et al., 2022). This convergence does not establish the validity of the findings; it indicates only that the same obstacles have been reported repeatedly across a decade of open-science policy development. On this basis, the study provides an up-to-date snapshot about the most and least common open science, data, and code sharing practices among reproducibility-engaged researchers, key barriers, and highlights the need for a systemic cultural shift within the research ecosystem.

### Survey-informed implications

**Journals:** On the side of journals, our results indicate that a simple requirement of code and/or data can improve computational reproducibility, as many studies are not too complex, and can be reproduced with relatively low effort, depending on the scientist’s computational background behind the attempt. Our results indicate also that journal requirements ranked first among motivating factors (score: 482, S8). This finding supports further consideration and evaluation of journal data- and code-availability policies, accompanied by appropriate guidance and exceptions where sharing is legally, ethically, or technically constrained.

**Researchers and authors:** Inadequate documentation ranked as the highest-rated barrier to computational reproducibility in our survey (score: 287, Q9, S5-S8). Accordingly, the findings support encouraging researchers to deposit code together with a basic set of metadata: dataset units, the sequence in which scripts should be run, the computational environment (operating system, package versions), and short descriptions of primary (raw) and processed data, in a README file. Relative to its effect on computational reproducibility, this is a low-cost measure. Guidance materials and training on tools that standardize computing environments (e.g., containerization and standardized OS platforms) would further support this practice in everyday research.

**Funders:** Funders could consider strengthening expectations for computational reproducibility in data management plans, including appropriate provisions for code and data. Given that 27.5% of respondents indicated that they had never attempted to reproduce another study, and that incentives and rewards ranked highly among the proposed supporting measures, targeted funding for replication and reproduction studies could also be evaluated.

**Institutions:** Research institutions and universities could consider integrating basic reproducibility skills (version control, structured data management, use of public repositories, e.g., Git, Docker, OSF, Zenodo) into mandatory doctoral training programs. Subgroup responses across career stages (S5–S7) may help inform the design of such training. Institutions could also develop promotion and evaluation criteria about data and code publications, replication and reproduction studies, and transparent reporting, that could be recognized alongside traditional publications.

These implications are interconnected. Journal requirements introduced without adequate institutional support place an undue burden on individual researchers; institutional reforms without funder backing lack sustainability. Effective change will require coordinated implementation across all stakeholder levels.

### Broader considerations on tooling and infrastructure

 The following considerations extend beyond what our survey can establish; they draw on the authors’ perceptions and literature, offering a context rather than as findings of this study.

Online platforms are convenient, but costly, and their computational capacity is tied to price tiers, which makes large-scale adoption unfeasible. In this context, local implementations of user interface (e.g., institutional level) like JupyterHub, RStudio Server or similar can be a leap in computational reproducibility. The implementation must be followed by sustained training campaigns to the end user. In a framework of research project: a light weight, cross-platform, plug-and-play (click-run-save-export) aimed at the end user, a docker-like for R, Python, and other common programming languages in data analyses, can be also a supportive solution. This is particularly relevant where researchers can be afraid to upload their data anywhere, due to sensitive data or other questions, even to the institutional servers, a local portable solution may be suitable.

### Future studies

Reproduction audits, which check whether the data and code behind published articles can actually be located, obtained, and re-executed, are the clearest next step. Such audits test adherence to FAIR principles ( Wilkinson et al., 2016) against practice and measure computational reproducibility directly, rather than relying on self-report, which tends to overstate what is available and reusable ( Trisovic et al., 2022). Audits alone, however, cannot show why reproduction fails. The barriers reported here, such as lack of time, publication pressure, funding, and documentation practices, leave no trace in a dataset or a code repository. Pairing an audit with a survey would connect what can be re-executed with the reasons behind it.

Future research could also build on this exploratory survey by employing larger, more systematically sampled populations to improve representativeness across disciplines and regions. Longitudinal studies would help capture whether awareness, incentives, and adoption of computational reproducibility practices change over time in response to new policies or infrastructures. Field-specific case studies could provide deeper insights into discipline-dependent barriers and enablers. Further work might also experimentally evaluate which interventions (e.g., institutional training programs, journal mandates) most effectively improve reproducibility in practice. Finally, integrating behavioral research on incentives with technical research on reproducibility infrastructure could clarify how cultural and technical factors interact in shaping researchers’ day-to-day workflows.

### Future studies

Reproduction audits, which check whether the data and code behind published articles can actually be located, obtained, and re-executed, are the clearest next step. Such audits test adherence to FAIR principles ( Wilkinson et al., 2016) against practice and measure computational reproducibility directly, rather than relying on self-report, which tends to overstate what is available and reusable ( Trisovic et al., 2022). Audits alone, however, cannot show why reproduction fails. The barriers reported here, such as lack of time, publication pressure, funding, and documentation practices, leave no trace in a dataset or a code repository. Pairing an audit with a survey would connect what can be re-executed with the reasons behind it.

Future research could also build on this exploratory survey by employing larger, more systematically sampled populations to improve representativeness across disciplines and regions. Longitudinal studies would help capture whether awareness, incentives, and adoption of computational reproducibility practices change over time in response to new policies or infrastructures. Field-specific case studies could provide deeper insights into discipline-dependent barriers and enablers. Further work might also experimentally evaluate which interventions (e.g., institutional training programs, journal mandates) most effectively improve reproducibility in practice. Finally, integrating behavioral research on incentives with technical research on reproducibility infrastructure could clarify how cultural and technical factors interact in shaping researchers’ day-to-day workflows.

## Conclusions

Digital tools play a crucial role in reproducible research by enabling standardization and automation; supporting data provenance and metadata tracking to ensure traceability and integrity; and facilitating transparent and shareable reporting. The survey revealed that barriers to computational reproducibility have remained largely unchanged, with the same challenges regarding data and code sharing already identified by Liu & Salganik (2019) and reported again by Reinecke et al. (2022). Issues such as inadequate documentation, incompatible computing environments, and unresolved software dependencies, as well as a lack of time, continue to hinder progress. This should be a key consideration and a focus on current and future metascience projects.

Practices to support computational reproducibility, such as the use of open-source software and open access publishing, were widely reported among the self-selected, open science-engaged respondents. However, this widespread awareness has not yet translated into the consistent implementation of more demanding reproducibility practices, such as study (pre) registration, replication efforts, or open peer review, which remain significantly underutilized. This awareness-implementation gap holds even within our reproducibility-engaged sample, underscoring that the obstacle is structural rather than a mere lack of awareness. Moreover, although most respondents agreed that data and code sharing are vital for scientific integrity and collaboration, self-reported sharing practices lag behind, with 45 of the 113 (40%) respondents reporting that they had not shared any data or code in the past five years.

These persistent challenges underscore the importance of providing effective technical support for researchers in the form of standardized tools, training, and methodological guidance to help overcome practical obstacles and to utilize computational methods more routinely. Training initiatives were identified as crucial for embedding good practices early in the research lifecycle. Embedding data management and data analysis into PhD, or even Bachelor’s (BS) and Master’s (MS) programs, is crucial across disciplines, as most of them increasingly rely on collecting, managing, and interpreting data.

A recurrent theme throughout the survey was the need for structural incentives and institutional support. Researchers claim that making work reproducible requires time, resources, and expertise; however, these efforts are rarely rewarded in outdated, conventional academic evaluation systems. Respondents also emphasized the role of journals, funding agencies, and institutions in promoting and rewarding open and reproducible research. These opinions highlight the need for the wider dissemination of new evaluation systems (DORA: Cagan, 2013; CoARA, 2022) that have not yet been widely applied.

In conclusion, even among researchers recruited through open science networks and predisposed toward these practices, where awareness of open science and reproducibility is high, widespread and consistent applications are still lacking. Adoption in the wider research community is unlikely to be higher. Addressing this gap requires coordinated efforts to remove technical barriers, redesign incentive structures, and create a culture supporting transparency and collaboration. The insights from this survey suggest that meaningful progress will depend not only on individual effort but also on systemic change across the research ecosystem.

## Software availability statement

For the analysis, Quarto ( Allaire et al., 2022) version 1.6.32 within RStudio ( Posit team, 2025) version 2025.05.0+496. R ( R Core Team, 2024) was used, that is an open-source scientific and technical publishing system, available at https://quarto.org/.

## Data Availability

Open Science Framework (OSF): 3.1. Computational reproducibility checks, Folder “Underlying_data”, https://doi.org/10.17605/OSF.IO/6YSRH, Gelsleichter et al., 2024. Folder, as .zip file, contains the following subfolders and files:
‐ Project root folder, contains all files for reproducibility, ‐ from conceptualization to analyses (Zip file) ‐ The 1, 2, 3 represents the chronological order of development o 1 Protocol_survey/
1. (Study protocol) o 2 Survey_documents_from_LimeSurvey/
1. Set of materials implemented in the LimeSurvey online platform
▪ form_and_questions/
▪ Original survey materials, exported from the LimeSurvey platform
▪ README - Computational reproducibility status survey.txt ▪
Supports overview of underlying files ▪
(other exported survey materials) ▪
survey_responses/ ▪
Raw anonymous responses, exported to several formats allowed ▪
by LimeSurvey platform ▪
README.txt ▪
(response data files) o 3 Survey_responses_analyses/
1. Analysis of survey responses (R with quarto) o
input/
1. Raw input files and other annexes used for the analysis o
script/
1. Complete analysis script to support reproducibility
2. with R language in Quarto environment o
rendered_output/
1. Result files generated from the Quarto document
▪*.docx
▪*.odt
▪*.pdf
▪*.pptx
▪*.epub
▪*.html
▪*.ipynb (Jupyter Notebook) Open Science Framework (OSF): 3.1. Computational reproducibility checks, Folder “Extended_data”, https://doi.org/10.17605/OSF.IO/6YSRH, Gelsleichter et al., 2024. Folder contains the following files: - S1_Study_protocol.pdf (also the informed consent page of the survey) https://osf.io/6ysrh/files/5ktsf - S2_Survey_questions.pdf https://osf.io/6ysrh/files/8du9x - S3_Responses_for_open_ended_questions_referred through_OSF.pdf https://osf.io/6ysrh/files/vca2w - S4_Cherries_checklist_filled.pdf https://osf.io/6ysrh/files/tqvdm - S5_Analyses_graphs_Jupyter_Notebook.ipynb (recommended for quick visualization directly within the OSF; high graphical resolution) https://osf.io/6ysrh/files/dgk2s - S6_Analyses_graphs_HTML.html (recommended for offline navigation; includes a table of contents sidebar and opens directly in any web browser) https://osf.io/6ysrh/files/gta6j - S7_Analyses_graphs_PDF.pdf (Use if.ipynb or.html files cannot be viewed) https://osf.io/6ysrh/files/az4qu - S8_Calculations_ranking.xlsx https://osf.io/6ysrh/files/69ufs Open Science Framework (OSF): 3.1. Computational reproducibility checks, Folder “Extended_data”, Checklist for ‘Survey about Barriers and Solutions for Enhancing Computational Reproducibility in Scientific Research’, file “S4_Cherries_checklist_filled.pdf”, https://doi.org/10.17605/OSF.IO/6YSRH, Gelsleichter et al., 2024. Data are available under the terms of the Creative Commons Attribution 4.0 International license (CC-BY 4.0). During the revision of this manuscript, the authors used ChatGPT (GPT-5.6 Sol, OpenAI; accessed July 2026) to assist with language editing and to improve the clarity and concision of the text. All AI-assisted suggestions were critically reviewed and edited by the authors, who take full responsibility for the final content of the manuscript.
