# Supplementary material for: Exploratory Insights into Barriers and Open Practices for Computational Reproducibility in Scientific Research
Source: F1000Res. 2026 Aug 11;14:1278. Originally published 2025 Nov 18. [Version 3] doi: 10.12688/f1000research.172013.3 (PMC13560004; doi:10.12688/f1000research.172013.3)
Supplement: Peer review history

# Reviewer report: Reviewer response for version 3

**DOI:** 10.5256/f1000research.204036.r509944

**Published:** 2026-08-26

**Authors:** Lex Bouter

**Affiliations:** 1 Amsterdam Universities Medical Center, Amsterdam, The Netherlands; 2 Philosophy, VU University Amsterdam Department of Philosophy (Ringgold ID: 404761), Amsterdam, North Holland, The Netherlands

I thank the authors for their thoughtful responses to the major and minor issues I raised. The revised manuscript is in my view substantially improved and is now suitable for being signed off. Hence, I approve the current version.

Is the work clearly and accurately presented and does it cite the current literature?

Partly

If applicable, is the statistical analysis and its interpretation appropriate?

Yes

Are all the source data underlying the results available to ensure full reproducibility?

Yes

Is the study design appropriate and is the work technically sound?

Partly

Are the conclusions drawn adequately supported by the results?

No

Are sufficient details of methods and analysis provided to allow replication by others?

Yes

Reviewer Expertise:

methodology, research integrity, open science, reproducibility, publication ethics, epidemiology

I confirm that I have read this submission and believe that I have an appropriate level of expertise to confirm that it is of an acceptable scientific standard.

# Reviewer report: Reviewer response for version 2

**DOI:** 10.5256/f1000research.197184.r473675

**Published:** 2026-04-21

**Authors:** Lex Bouter

**Affiliations:** 1 Amsterdam Universities Medical Center, Amsterdam, The Netherlands; 2 Philosophy, VU University Amsterdam Department of Philosophy (Ringgold ID: 404761), Amsterdam, North Holland, The Netherlands

The manuscript describes the findings of a survey among 254 scholars (of whom 133 answered all questions) on several aspects of computational reproducibility. It reports on the prevalence of self-reported open science and reproducibility practices and perceived barriers and stimulating factors to engaging in these practices. The manuscript is reasonably clearly written and the graphs are very informative. The authors did a good job regarding transparency (open methods, open data and open codes). Version 2 is the first version I saw, as was not involved in the first review round. Although I’ve read both earlier review reports and the rebuttal by the authors, I feel free to raise new points because this is my first chance to raise them.

**Major concerns**

- The Introduction section does not explain why the topic is studied in a survey. While surveys are a good method to sample perceptions and opinions, they are much less suitable to study actual behaviour. What people report to have done, often differs from what they actually did. It has been well documented that when scholars claim to have engaged in FAIR open data an open codes, this often turns out to be not at all the case. Please explain why you did not check whether data sets and codes could actually be obtained and successfully re-used?
- The authors admit that the recruitment was highly selective, because it focussed on scholars involved in reproducibility and open science. This is illustrated by the high prevalences of open science and reproducibility practices, like 52% reporting to engage in Registered Reports, which is much more than what one would expect in an average sample of researchers. Please flag this much clearer, including in the abstract. In my opinion the main conclusion of the report should be that open science and reproducibility aficionados don’t always practice what they preach. Also the barriers and stimulating factors likely mainly reflect the views of this self-selected group of researchers.
- The convenience sampling strategy through social media, blogs and newsletters, implies that no response rate could be calculated and selection bias could not be explored. The consequence is that, also for the target group of open science and reproducibility aficionados, the generalizability of the findings is unknown. This is another study limitation that should be better spelled out in the manuscript. It is flagged, but strangely (and wrongly) it is suggested that the fact that some demographic characteristics are reported softens the selectivity of the sample studied.
- Even when the demographics would be exactly the same as for the average population of scholars, the probable selection on enthusiasm for open science and reproducibility would render the survey results uninformative about that average researcher population. The fact that studies with similar selection biases show similar findings does not mean that these findings are valid.

**Minor concerns**

- The Results section is difficult to read, because it’s mainly a list of numerical details of the findings. I believe this should be transferred to a digital supplement. In the main text a narrative summary, highlighting the most important findings shown in the figures, would be the better approach.
- Please tone down the list of recommendations based on this small survey among a selected population. In fact you go far beyond what was investigated. I’m not saying that the recommendations make no sense, because they do. But most could equally well be made without this survey.
- I’m quite puzzled by the fact that you don’t recommend future studies that actually check publications for FAIR open data and open codes and actually investigate whether computational reproducibility is possible and successful.
- I believe that the manuscript can and should be shortened substantially. Less is more.

Is the work clearly and accurately presented and does it cite the current literature?

Partly

If applicable, is the statistical analysis and its interpretation appropriate?

Yes

Are all the source data underlying the results available to ensure full reproducibility?

Yes

Is the study design appropriate and is the work technically sound?

Partly

Are the conclusions drawn adequately supported by the results?

No

Are sufficient details of methods and analysis provided to allow replication by others?

Yes

Reviewer Expertise:

methodology, research integrity, open science, reproducibility, publication ethics, epidemiology

I confirm that I have read this submission and believe that I have an appropriate level of expertise to state that I do not consider it to be of an acceptable scientific standard, for reasons outlined above.

# Response

**Published:** 2026-07-27

**Authors:** Monika Varga

**Affiliations:** Institute of Animal Sciences, Magyar Agrar- es Elettudomanyi Egyetem - Kaposvari Campus, Kaposvár, Somogy County, Hungary

*The manuscript describes the findings of a survey among 254 scholars (of whom 133 answered all questions) on several aspects of computational reproducibility. It reports on the prevalence of self-reported open science and reproducibility practices and perceived barriers and stimulating factors to engaging in these practices. The manuscript is reasonably clearly written and the graphs are very informative. The authors did a good job regarding transparency (open methods, open data and open codes). Version 2 is the first version I saw, as was not involved in the first review round. Although I’ve read both earlier review reports and the rebuttal by the authors, I feel free to raise new points because this is my first chance to raise them.*

 We thank Dr. Bouter for his careful reading and for his assessment of the manuscript's clarity, the figures, and the transparency of the methods, data, and code. We also appreciate that, although he joined at the second round, he raised these new points; they are substantive and have materially improved the paper. We respond to each concern below and adressed these changes to the manuscript.

*Major concerns*

*The Introduction section does not explain why the topic is studied in a survey. While surveys are a good method to sample perceptions and opinions, they are much less suitable to study actual behaviour. What people report to have done, often differs from what they actually did. It has been well documented that when scholars claim to have engaged in FAIR open data an open codes, this often turns out to be not at all the case. Please explain why you did not check whether data sets and codes could actually be obtained and successfully re-used?*

 We agree that self-reported practices may differ from actual conduct and appreciate the Reviewer’s important point, and we have taken this into account.

 On the choice of a survey: our research questions concern researchers' perceptions, attitudes, perceived barriers (e.g., lack of time, pressure to publish), needs, and self-reported practices regarding computational reproducibility, subjective constructs that cannot be obtained by inspecting published data sets and codes. A survey is the appropriate instrument for exploring researchers’  reported reasons for adopting or not adopting reproducible practices,  as well as their perceptions of what might facilitate their adoption. It also enabled us to reach respondents across a broad range of disciplines and geographical regions. For example, survey revealed that researchers in lower-GDP countries rely more on paper than those in higher-GDP settings (see Extended data S5-S6-S7, Q22), which an audit of published artifacts would not capture. We have added a brief statement of this rationale to the Abstract, Introduction and now label behavioural items explicitly as self-reported across Methods, Results, Discussion, Recommendations and Conclusions.

 Another important aspect is we ultimately decided to run a fully anonymous survey, keeping privacy and avoiding spamming researchers. Because the survey was fully anonymous (no cookies, no IP address collection, no identifiers), social desirability pressures are reduced, although not eliminated.

*The authors admit that the **recruitment was highly selective**, because it focussed on scholars involved in reproducibility and open science. This is illustrated by the high prevalences of open science and reproducibility practices, like 52% reporting to engage in Registered Reports, which is much more than what one would expect in an average sample of researchers. **Please flag this much clearer, including in the abstract.** In my opinion the main conclusion of the report should be that open science and reproducibility aficionados don’t always practice what they preach. Also the barriers and stimulating factors likely mainly reflect the views of this self-selected group of researchers.*

*T **he convenience sampling strategy** through social media, blogs and newsletters, implies that no response rate could be calculated and selection bias could not be explored. The consequence is that, also for the target group of open science and reproducibility aficionados, the generalizability of the findings is unknown. This **is another study limitation that should be better spelled out in the manuscript.** It is flagged, but strangely (and wrongly) it is suggested that the fact that some demographic characteristics are reported softens the selectivity of the sample studied.*

*Even when the demographics would be exactly the same as for the average population of scholars, the probable selection on enthusiasm for open science and reproducibility would render the survey results uninformative about that average researcher population. The fact that studies with similar selection biases show similar findings does not mean that these findings are valid.*

 We agree with the reviewer that our open recruitment strategy resulted in a self-selected sample and limits generalizability of the findings. When designing the study, we considered the trade-offs associated with different recruitment strategies. Direct, list-based recruitment can introduce coverage biases, for example with respect to career stage, language, and geographical region; countries with larger and better-resourced research systems may be disproportionately represented, while regions such as Latin America and Africa may remain underrepresented. It would also require access to and maintenance of a database containing researchers’ contact details. Open recruitment avoids the need for such a database and can broaden disciplinary and geographic reach, but it cannot control who self-selects into the study. We accepted this trade-off, prioritizing breadth and full anonymity. The resulting sample included respondents from several disciplinary fields, with a relatively even distribution acrossNatural sciences (32), Medical (28), Agricultural (27), Engineering & Technology (26), Social Sciences & Humanities (20) among the 133 respondents. Survey also reached respondents in lower-research-output settings, although we recognize that the geographical distribution remained uneven.

 We acknowledge the limitations that the reviewer identifies. Disciplinary breadth does not establish representativeness within disciplines; and as a response rate could not be calculated, we cannot characterize non-respondents or quantify selection bias. We accept that this constrains what the findings can support. The findings should therefore be interpreted as reflecting the perceptions and self-reported practices of a self-selected and likely reproducibility-aware group of researchers, rather than those of the broader researcher population. Nevertheless, this group is particularly relevant in understanding the perceived barriers that may prevent even researchers who are aware of, or interested in reproducibility from consistently adopting reproducible practices.

 Despite this was present in the Material and Methods section (under “Survey population and recruitment”) the issue is now flagged in the Abstract and has been substantially explained in the Material and Methods, Results (as ‘reported’), Discussion (as ‘reported’), Limitations, and reflected in the Conclusions. We have also consistently described the findings in the Results and Discussion as “reported” or “self-reported” and have removed the passages that implied demographic reporting or agreement with previous studies mitigates selection bias.

 One clarification regarding the illustrative figure: 52% refers to respondents who reported never using registered reports; the corresponding engagement figure is its complement, 48% reporting at least occasional use, distributed across 18% rarely, 18% sometimes, 9% frequently, and 3% always, out of 120 respondents. The original sentence in the Abstract was ambiguously worded and has been rephrased. The reviewer's point is unaffected: 48% remains far above what would be expected in an unselected sample, and we now use this figure explicitly as an indicator of selectivity.

*Minor concerns*

*The Results section is difficult to read, because it’s mainly a list of numerical details of the findings. I believe this should be transferred to a digital supplement. In the main text a narrative summary, highlighting the most important findings shown in the figures, would be the better approach.*

*Please tone down the list of recommendations based on this small survey among a selected population. In fact you go far beyond what was investigated. I’m not saying that the recommendations make no sense, because they do. But most could equally well be made without this survey.*

*I’m quite puzzled by the fact that you don’t recommend future studies that actually check publications for FAIR open data and open codes and actually investigate whether computational reproducibility is possible and successful.*

*I believe that the manuscript can and should be shortened substantially. Less is more.*

 Some of the reviewer’s requests concerning the level of detail and the recommendations differed from those made during the first review round. We therefore sought to balance the two sets of comments while also improving the manuscript’s focus and concision.

 On the Results section, we agree that it is dense and numerical. In this revision we moved the most granular material, the explicit weighted-score calculations to the supplement (S8), keeping only the resulting scores in the main text. The first-round reviewers, however, asked for more explicit reporting of subgroup comparisons (early-career vs. senior researchers, Europe vs. non-Europe); we accommodated those requests without adding figures by pointing to the Extended data (supplementary materials S5-S7). The narrative synthesis was also strengthened in the Discussion, where each subsection opens with a "Key message" paragraph summarizing the main finding in plain language before any numbers are discussed. We believe this arrangement balances readability with the completeness requested in the first round.

 On the recommendations, the point is well taken. Here too, the first round of review led us in the opposite direction, asking for a more structured, stakeholder-specific set; rather than removing this section entirely, we have shortened and reframed it as “Survey-informed implications.” We retained only implications linked to specific survey findings, softened prescriptive language, and explicitly stated that these implications represent priorities for consideration and further evaluation rather than interventions whose effectiveness was established by the survey. Broader considerations based on the literature and the authors’ experience are presented separately and explicitly identified as extending beyond what the survey can establish.

 The "Future studies" section was restructured to place reproduction audits, which test in practice whether data and code are findable and reusable and whether published analyses can be re-executed, as the priority for future work.

 On the overall length, we understand the reviewer's preference for a shorter paper. The current length reflects material added in response to the two review rounds: terminology definitions and recent literature (round 1), subgroup breakdowns (round 1), and the additions requested in this round. A substantial reduction would have required removing material introduced in response to the earlier reviewers. Nevertheless, we reviewed the manuscript throughout thoroughly for concision: we removed repeated numerical descriptions, transferred detailed calculations to the Extended data, condensed several Discussion subsections, and streamlined and reframed the recommendations. We believe that the revised manuscript is now more focused while retaining the information necessary to address the comments raised during both review rounds.

# Reviewer report: Reviewer response for version 2

**DOI:** 10.5256/f1000research.197184.r473257

**Published:** 2026-04-09

**Authors:** Sheeba Samuel

**Affiliations:** 1 Technische Universitat Chemnitz, Chemnitz, Saxony, Germany

Thank you for the thorough revisions. The authors have addressed the comments and suggestions from both reviewers, and the revisions significantly strengthen the manuscript. To further enhance the quality of the work, the authors should ensure their practical recommendations are more explicitly aligned and grounded in state-of-the-art research.

Is the work clearly and accurately presented and does it cite the current literature?

Partly

If applicable, is the statistical analysis and its interpretation appropriate?

Yes

Are all the source data underlying the results available to ensure full reproducibility?

Yes

Is the study design appropriate and is the work technically sound?

Yes

Are the conclusions drawn adequately supported by the results?

Yes

Are sufficient details of methods and analysis provided to allow replication by others?

Yes

Reviewer Expertise:

Computer Science, Reproducible Research, Data Provenance, Semantic Web, Knowledge Graphs

I confirm that I have read this submission and believe that I have an appropriate level of expertise to confirm that it is of an acceptable scientific standard.

# Reviewer report: Reviewer response for version 1

**DOI:** 10.5256/f1000research.189689.r442319

**Published:** 2026-01-22

**Authors:** Frank Olaf Ostermann

**Affiliations:** 1 University of Twente, Enschede, The Netherlands

A good thing about open peer review is that reviews can refer to a previous one. In this case, the first review has already done a remarkable job at evaluating the paper. In fact, there is no statement I would disagree with, and several statements that I strongly support. My review will thus briefly state those I particularly consider important and then try to add a few additional points from my perspective. 

 But first, I want to emphasize that the authors have done a commendable job at making their own work open and reproducible. There is extensive information on all aspects of the study available. 

 However, the paper itself is indeed quite exploratory in nature, and a different study design could allow more systematic and inferential analysis that goes beyond the descriptive statistics that are reported. On a sidenote, I could not find information on the members of the consortium that designed the survey - were they sufficiently diverse (backgrounds, career stages, ...) to replace a pilot study with respondent feedback? 

 I also view the lack of systematic definition of terminology as problematic - given the variety of terminology surrounding open and reproducible research (reproduction, replication, generalisation, robustness, reanalysis, repeatability, ...), it seems necessary to clarify the authors' use of these terms and their use in the survey.

 The referenced literature is not a strong point of this paper, which refers to "recent studies" several times, but then these studies are from more than 10 years ago. While of course newer literature is not always more relevant, in the context of open and reproducible research, recency matters. The reproducibility crisis was 'diagnosed' only two decades ago, with many papers during the following ten years or so trying to determine the scope of the problem. It's been only during the last decade that research proposing and evaluating solutions has become more prominent, and such works would be very relevant for this paper. There is a hint that the paper (or at least parts of it) has been in the works for some time and might need an update: it refers to studies from 2018 and 2019 as being "five years ago". I've also wondered whether there was a second round of literature review after the survey. The open survey questions might contain valuable hints at more recent studies, worthy following up. In any case, additional insights would be possible with a more systematic comparison with other surveys. From my own discipline, which is admittedly a smaller one and thus not often popping up in cross-disciplinary research, I could cite (Kedron et al.,2024) as well as my own work. 

 Lastly, the interpretation and discussion of the survey outcomes could be more assertive. For example, the repeated claim that making work more reproducible requires significant time and effort and thus merits additional rewards - I think this needs some contextualization and critical appraisal. A basic level or reproducibility does not require that much effort or skill: non-proprietary software, structured documentation, and a deposition of code and data on a public repository. For most of the research that I have been reviewing over the past 15 years, achieving this basic level of reproducibility would not have been too hard. But maybe the survey respondents tried to provide an answer for a statement that is seems to be missing in the survey: Funding, rewards, and recognition for reproducing and replicating studies. Because that is the final goal, why we should make studies more reproducible: so that eventually there is a documented/published attempt at reproduction or replication. If only a tiny fraction of all output ever actually gets reproduced, then the question of why to spend even moderate effort on making something reproducible is a valid concern. This important perspective is only discussed in passing in a very short section.

Is the work clearly and accurately presented and does it cite the current literature?

Partly

If applicable, is the statistical analysis and its interpretation appropriate?

Not applicable

Are all the source data underlying the results available to ensure full reproducibility?

Yes

Is the study design appropriate and is the work technically sound?

Yes

Are the conclusions drawn adequately supported by the results?

Yes

Are sufficient details of methods and analysis provided to allow replication by others?

Yes

Reviewer Expertise:

geographic information science, geodata engineering, participatory research, open and reproducible research

I confirm that I have read this submission and believe that I have an appropriate level of expertise to confirm that it is of an acceptable scientific standard, however I have significant reservations, as outlined above.

## References

[1] : Reproducible Research Practices and Barriers to Reproducible Research in Geography: Insights from a Survey. *Annals of the American Association of Geographers* .2024;114(2) : 10.1080/24694452.2023.2276115369-386 10.1080/24694452.2023.2276115

# Response

**Published:** 2026-03-02

**Authors:** Monika Varga

**Affiliations:** Institute of Animal Sciences, Magyar Agrar- es Elettudomanyi Egyetem - Kaposvari Campus, Kaposvár, Somogy County, Hungary

Dear Dr. Frank Olaf Ostermann,

 We are grateful for your insightful comments and for drawing our attention to areas requiring further elaboration. Your feedback has considerably improved the quality of our work. Below please find our point-by-point reply.

 *A good thing about open peer review is that reviews can refer to a previous one. In this case, the first review has already done a remarkable job at evaluating the paper. In fact, there is no statement I would disagree with, and several statements that I strongly support. My review will thus briefly state those I particularly consider important and then try to add a few additional points from my perspective.

 But first, I want to emphasize that the authors have done a commendable job at making their own work open and reproducible. There is extensive information on all aspects of the study available.

*Thank you for the observation! Appreciated!*

 *However, the paper itself is indeed quite exploratory in nature, and a different study design could allow more systematic and inferential analysis that goes beyond the descriptive statistics that are reported. On a sidenote, I could not find information on the members of the consortium that designed the survey - were they sufficiently diverse (backgrounds, career stages, ...) to replace a pilot study with respondent feedback?

*Thank you for the observation!*

*We have reframed the manuscript as an exploratory survey throughout the text. We clarified the scope and addressed representativeness concerns.*

*Also, the survey was designed by a diverse group of scientists from Agronomy, IT, Geomatics to Medical field, PhD to Senior researchers. Regarding the pilot, aside of technical aspects we collect feedback on survey length, typo, logical aspects, dropping and inclusion of questions.*

*These points were addressed in the text.*

 *I also view the lack of systematic definition of terminology as problematic - given the variety of terminology surrounding open and reproducible research (reproduction, replication, generalisation, robustness, reanalysis, repeatability, ...), it seems necessary to clarify the authors' use of these terms and their use in the survey.

*Thank you for the observation!*

*The concept of ‘Computational reproducibility’ was deeper explored and detailed. Also, the definition of ‘Replication’ was also provided. Most of the survey questions gravitated around reproducibility, however pointing to computational reproducibility. As the tooltip (pop-up) tool in the survey platform was available. The definition of Replication studies was also present in the survey.*

 *The referenced literature is not a strong point of this paper, which refers to "recent studies" several times, but then these studies are from more than 10 years ago. While of course newer literature is not always more relevant, in the context of open and reproducible research, recency matters. The reproducibility crisis was 'diagnosed' only two decades ago, with many papers during the following ten years or so trying to determine the scope of the problem. It's been only during the last decade that research proposing and evaluating solutions has become more prominent, and such works would be very relevant for this paper. There is a hint that the paper (or at least parts of it) has been in the works for some time and might need an update: it refers to studies from 2018 and 2019 as being "five years ago". I've also wondered whether there was a second round of literature review after the survey. The open survey questions might contain valuable hints at more recent studies, worthy following up. In any case, additional insights would be possible with a more systematic comparison with other surveys. From my own discipline, which is admittedly a smaller one and thus not often popping up in cross-disciplinary research, I could cite (Kedron et al.,2024) as well as my own work.

*Thank you for the observation!*

*By the time we wrote the protocol and first draft (end of 2023), the time span was the above mentioned. Thank you also for enlightenment with this citation, we have used gathering academic search tools for papers and these ones were, yet, left out. Both currently adjusted in the text, as eight studies were added. Some examples of peer-reviewed studies include Gomes et al. (2022); Trisovic et al. (2022); Nosek et al. (2022); and Wilkinson et al. (2016). We also incorporate Kedron et al. (2024) and Cerutti et al. (2021), as suggested by the reviewer.*

 *Lastly, the interpretation and discussion of the survey outcomes could be more assertive. For example, the repeated claim that making work more reproducible requires significant time and effort and thus merits additional rewards - I think this needs some contextualization and critical appraisal. A basic level or reproducibility does not require that much effort or skill: non-proprietary software, structured documentation, and a deposition of code and data on a public repository. For most of the research that I have been reviewing over the past 15 years, achieving this basic level of reproducibility would not have been too hard. But maybe the survey respondents tried to provide an answer for a statement that is seems to be missing in the survey: Funding, rewards, and recognition for reproducing and replicating studies. Because that is the final goal, why we should make studies more reproducible: so that eventually there is a documented/published attempt at reproduction or replication. If only a tiny fraction of all output ever actually gets reproduced, then the question of why to spend even moderate effort on making something reproducible is a valid concern. This important perspective is only discussed in passing in a very short section.

*Thank you for the observation!*

*We are grateful for this incisive critique, which touches on a genuinely important conceptual distinction that our manuscript had not sufficiently developed. We agree that the claim that reproducibility requires "significant effort" warrants critical examination. We have now added a paragraph to address this concern. We thank the reviewer for bringing this perspective; it sharpens the practical contribution of the paper.*

# Reviewer report: Reviewer response for version 1

**DOI:** 10.5256/f1000research.189689.r439988

**Published:** 2025-12-30

**Authors:** Sheeba Samuel

**Affiliations:** 1 Technische Universitat Chemnitz, Chemnitz, Saxony, Germany

The article reports the results of a multidisciplinary, international survey examining barriers, practices, and potential solutions related to computational reproducibility in scientific research. Using an anonymous multidisciplinary survey with 133 complete responses (mostly from Europe), the authors investigate the adoption of open science practices, existing barriers to code and data sharing, and potential solutions to enhance reproducibility. The results indicate high awareness and relatively widespread adoption of open-source software and open-access publishing, contrasted with low uptake of more demanding practices such as preregistration, registered reports, replication studies, and open peer review. Across domains, lack of time, pressure to publish, insufficient funding, and inadequate documentation emerge as the most prominent barriers to computational reproducibility. Respondents strongly emphasize the role of journals, institutions, and incentive structures in driving change.

 Strengths

 * Computational reproducibility is a central concern across scientific disciplines, and the study provides an up-to-date snapshot that complements and extends earlier survey-based research in this area.

 * The inclusion of respondents from different disciplines enhances the breadth of perspectives and supports cross-disciplinary comparisons.

 * The study protocol, survey instrument, anonymized data, and analysis scripts are openly available via OSF.

 * The combination of closed-ended questions with open-ended responses provides both quantitative trends and qualitative insights, adding depth to the interpretation of barriers and proposed solutions.

 Weaknesses

 * The respondent pool is heavily skewed towards Europe (80%), which limits the global representativeness of the findings, particularly for researchers in developing nations or different institutional cultures.

 * With only 133 complete responses from an initial 254 (and a likely low overall response rate given the social media recruitment), the study may suffer from self-selection bias, where researchers already interested in open science are more likely to participate. While the survey was initiated by many participants, only 133 complete responses were analyzed, which constrains subgroup analyses (e.g., by discipline, region, or career stage). The large number of questions (35 across multiple sections) likely contributed to dropout after the demographic section and may have affected response quality for later items.

 * The data is based on researchers' perceptions of their own practices. There may be a gap between what researchers claim to do and their actual day-to-day adherence to rigorous reproducibility standards. For example, High levels of agreement with statements framing open science practices as “good research practice” may reflect normative expectations rather than actual behavior.

 * The analysis relies almost entirely on descriptive statistics. While appropriate for an exploratory survey, more inferential or comparative analyses (even exploratory ones) could strengthen the contribution.

 Improvements

 * While the authors appropriately acknowledge limitations related to sampling and response rate, the manuscript would benefit from a clearer and more explicit framing of the study as exploratory rather than representative. Strengthening the language around what can and cannot be generalized to the broader research community would help readers interpret the findings more accurately.

 * The main manuscript would benefit from more explicit comparisons across career stages, disciplines, or regions (e.g., early-career vs. senior researchers, Europe vs. non-Europe). Even exploratory comparisons could yield actionable insights.

 * It is not sufficiently clear how the results of this study differ from those of earlier surveys on reproducibility, data sharing, or computational practices. A more explicit comparison with prior studies (e.g. what has changed, what remains unchanged, and what new patterns emerge) would strengthen the contribution and help readers understand the added value of this work. Literature review could be improved.

 * The motivation for conducting this survey should also be stated more clearly and earlier in the manuscript. While the introduction references the reproducibility “crisis” and prior literature, the specific gap that this study aims to fill—beyond providing an updated snapshot—remains somewhat implicit.

 * the decision to keep the survey open from 23.02.2024 to 30.09.2024 requires clarification. The manuscript does not explain why this relatively long data collection period was chosen, nor how it may have influenced participation, response consistency, or seasonal effects.

 * The paper occasionally uses terms related to reproducibility and replication interchangeably, which can be confusing in a technical survey and may be interpreted differently by respondents (Same applies for  other concepts). Providing brief, standardized definitions in the main text could improve conceptual clarity and consistency.

 * The demographic reporting of 254 respondents has been mentioned but not the 133 participants. This should be clarified to avoid confusion and to ensure that readers understand which population the results actually describe.

 * While the conclusions highlight the need for systemic change, the manuscript could benefit from a more structured set of recommendations targeted at specific stakeholders (e.g., journals, institutions, funders, training programs). Clear, prioritized action points would enhance the practical impact of the findings.

 * Given the survey’s distribution through open science networks, respondents are likely more aware of and favorable toward reproducibility practices. A more explicit discussion of how this self-selection may inflate reported awareness or positive attitudes—and how this affects interpretation—would strengthen the methodological transparency.

 * The current title may overstate the study's outcomes by promising 'Solutions.' Since the data captures researcher viewpoints rather than validated interventions, the authors should rename the article to reflect its exploratory nature.

 * The manuscript could briefly outline how future research might build on this work.

Is the work clearly and accurately presented and does it cite the current literature?

Partly

If applicable, is the statistical analysis and its interpretation appropriate?

Yes

Are all the source data underlying the results available to ensure full reproducibility?

Yes

Is the study design appropriate and is the work technically sound?

Yes

Are the conclusions drawn adequately supported by the results?

Yes

Are sufficient details of methods and analysis provided to allow replication by others?

Yes

Reviewer Expertise:

Computer Science, Reproducible Research, Data Provenance, Semantic Web, Knowledge Graphs

I confirm that I have read this submission and believe that I have an appropriate level of expertise to confirm that it is of an acceptable scientific standard, however I have significant reservations, as outlined above.

# Response

**Published:** 2026-03-02

**Authors:** Monika Varga

**Affiliations:** Institute of Animal Sciences, Magyar Agrar- es Elettudomanyi Egyetem - Kaposvari Campus, Kaposvár, Somogy County, Hungary

Dear Dr. Sheeba Samuel,

 We appreciated your effort to highlight edges where we need to draw more attention. Your comments are added great value for the publication. Below please find a point-by-point reply.

 * While the authors appropriately acknowledge limitations related to sampling and response rate, the manuscript would benefit from a clearer and more explicit framing of the study as exploratory rather than representative. Strengthening the language around what can and cannot be generalized to the broader research community would help readers interpret the findings more accurately.

*Thank you for the observation!*

*We have reframed the manuscript as an exploratory survey throughout the text. We have clarified the scope and addressed representativeness concerns.*

 * The main manuscript would benefit from more explicit comparisons across career stages, disciplines, or regions (e.g., early-career vs. senior researchers, Europe vs. non-Europe). Even exploratory comparisons could yield actionable insights.

*Thank you for the observation!*

*The comparisons across career stages, disciplines, developed and developing countries, were added through the text, across several questions. Supporting document with figures is placed in “Extended data” under S5, S6, S7.*

 * It is not sufficiently clear how the results of this study differ from those of earlier surveys on reproducibility, data sharing, or computational practices. A more explicit comparison with prior studies (e.g. what has changed, what remains unchanged, and what new patterns emerge) would strengthen the contribution and help readers understand the added value of this work. Literature review could be improved.

*Thank you for the observation!*

*The discussion was expanded comparing with other studies, eight peer-reviewed studies were added, including Gomes et al. (2022); Trisovic et al. (2022); Nosek et al. (2022); and Wilkinson et al. (2016). We also incorporate Kedron et al. (2024) and Cerutti et al. (2021), as suggested by the other reviewer. The discussion covers: what has changed (open science infrastructure, funder mandates, platform maturity), what has remained the same (time barriers, documentation gaps), and what new patterns emerge (cross-disciplinary consistency in behavioral inertia despite policy progress).*

 * The motivation for conducting this survey should also be stated more clearly and earlier in the manuscript. While the introduction references the reproducibility “crisis” and prior literature, the specific gap that this study aims to fill—beyond providing an updated snapshot—remains somewhat implicit.

*Thank you for the observation!*

*The motivation-related section was expanded, and the goals was further detailed.*

 * the decision to keep the survey open from 23.02.2024 to 30.09.2024 requires clarification. The manuscript does not explain why this relatively long data collection period was chosen, nor how it may have influenced participation, response consistency, or seasonal effects.

*Thank you for the observation!*

*As per the study protocol, the survey was designed to be online for ~three months (from 23.02.2024 to 31.05.2024), however as the number of respondents still limited it was decided to extend until 30.09.2024, spanning ~six months. This was clarified in the manuscript.*

 * The paper occasionally uses terms related to reproducibility and replication interchangeably, which can be confusing in a technical survey and may be interpreted differently by respondents (Same applies for  other concepts). Providing brief, standardized definitions in the main text could improve conceptual clarity and consistency.

*Thank you for the observation!*

*The concept of ‘Computational reproducibility’ was deeper explored and detailed. Also, the definition of ‘Replication’ was also provided. Most of the survey questions gravitated around reproducibility, however pointing to computational reproducibility. As the tooltip (pop-up) tool in the survey platform was available. The definition of Replication studies was also present in the survey.*

 * The demographic reporting of 254 respondents has been mentioned but not the 133 participants. This should be clarified to avoid confusion and to ensure that readers understand which population the results actually describe.

*Thank you for the observation!*

*The respective participants’ section was expanded to distinguish between: 254 initial respondents (survey initiated), 194 who completed demographics and 133 who completed the full survey (final analytical sample). This breakdown addresses potential confusion regarding dropouts and sample composition. For clarity, we have indicated the current values in each analysis.*

 * While the conclusions highlight the need for systemic change, the manuscript could benefit from a more structured set of recommendations targeted at specific stakeholders (e.g., journals, institutions, funders, training programs). Clear, prioritized action points would enhance the practical impact of the findings.

*Thank you for the observation!*

*We agree that actionable recommendations strengthen the practical impact of the work. We have expanded the "Study limitations and recommendations" section with a structured set of prioritized recommendations targeting journals, institutions, funders, and training programs, grounded directly in our survey findings. We have also noted the interdependence of these recommendations to avoid the impression that any single-actor intervention is sufficient.*

 * Given the survey’s distribution through open science networks, respondents are likely more aware of and favorable toward reproducibility practices. A more explicit discussion of how this self-selection may inflate reported awareness or positive attitudes—and how this affects interpretation—would strengthen the methodological transparency.

*Thank you for the observation!*

*We thank the reviewer for raising this important methodological point. We have substantially expanded the discussion of self-selection bias. Also reframed this limitation constructively: even among a motivated and (potential) self-selected group, substantial barriers persist, which is itself an informative finding for policy. A brief additional note on this point has been added to the limitations section.*

 * The current title may overstate the study's outcomes by promising 'Solutions.' Since the data captures researcher viewpoints rather than validated interventions, the authors should rename the article to reflect its exploratory nature.

*Thank you for the observation!*

*The title was redesigned, ‘solutions’ was replaced by ‘insights’, and ‘exploratory was added.*

 * The manuscript could briefly outline how future research might build on this work.

*Thank you for the observation!*

*A paragraph outlining optional future work was added. Specifically, we highlight opportunities for larger and more representative samples, longitudinal monitoring of changes in reproducibility practices, to determine which measures most effectively improve computational reproducibility.*
